# Comparative Genomic Analysis of Antarctic *Pseudomonas* Isolates with 2,4,6-Trinitrotoluene Transformation Capabilities Reveals Their Unique Features for Xenobiotics Degradation

**DOI:** 10.3390/genes13081354

**Published:** 2022-07-28

**Authors:** Ma. Ángeles Cabrera, Sebastián L. Márquez, José M. Pérez-Donoso

**Affiliations:** Center for Bioinformatics and Integrative Biology (CBIB), Facultad Ciencias de la Vida, Universidad Andrés Bello, Santiago 8320000, Chile; mac.biosc@gmail.com (M.Á.C.); sebastian.marquez@usach.cl (S.L.M.)

**Keywords:** *Pseudomonas*, TNT, comparative genomics, Antarctica, bioremediation, xenobiotics

## Abstract

The nitroaromatic explosive 2,4,6-trinitrotoluene (TNT) is a highly toxic and persistent environmental pollutant. Since physicochemical methods for remediation are poorly effective, the use of microorganisms has gained interest as an alternative to restore TNT-contaminated sites. We previously demonstrated the high TNT-transforming capability of three novel *Pseudomonas* spp. isolated from Deception Island, Antarctica, which exceeded that of the well-characterized TNT-degrading bacterium *Pseudomonas putida* KT2440. In this study, a comparative genomic analysis was performed to search for the metabolic functions encoded in the genomes of these isolates that might explain their TNT-transforming phenotype, and also to look for differences with 21 other selected pseudomonads, including xenobiotics-degrading species. Comparative analysis of xenobiotic degradation pathways revealed that our isolates have the highest abundance of key enzymes related to the degradation of fluorobenzoate, TNT, and bisphenol A. Further comparisons considering only TNT-transforming pseudomonads revealed the presence of unique genes in these isolates that would likely participate directly in TNT-transformation, and others involved in the β-ketoadipate pathway for aromatic compound degradation. Lastly, the phylogenomic analysis suggested that these Antarctic isolates likely represent novel species of the genus *Pseudomonas*, which emphasizes their relevance as potential agents for the bioremediation of TNT and other xenobiotics.

## 1. Introduction

Owing to their high chemical stability and low degradability, nitroaromatic pollutants are extremely difficult to remove from the environment, and because of their extensive use and disposal by both military and civilian activities, they are now widely distributed both in soils and waters, becoming a major environmental concern [1]. One of the most toxic and recalcitrant compounds among nitroaromatics is 2,4,6-trinitrotoluene (TNT), a highly noxious, mutagenic, and deleterious xenobiotic. Due to its great explosive power, it is mainly used for military purposes, but also as a valuable resource in civil engineering activities, mining, and propulsion technologies [2,3,4,5]. Owing to its negative effects on health, it has been classified as a hazardous substance by the United States Environmental Protection Agency (US EPA) [6]. Although diverse physical and chemical methods have been developed for removing TNT from the environment, they have not been useful enough, mainly due to their high cost and the generation of toxic by-products [7]. In contrast, bioremediation, a biological approach to remove toxic waste by using biological agents such as microorganisms, offers advantages to achieve complete transformation or mineralization of TNT in an environmentally friendly manner [3]. However, the development of effective treatments and strategies for complete onsite clean-up and detoxification of this compound is an issue that remains unsolved and has regained research interest in recent years [8,9,10,11].

Microorganisms have evolved to thrive in virtually every environment on Earth, using all kinds of compounds as a source of energy, and nitroaromatics are not an exception. Bacteria from several genera, such as *Escherichia*, *Enterobacter*, *Citrobacter*, *Clostridium*, *Desulfovibrio*, *Klebsiella*, and *Pseudomonas* (among some others), have been reported so far as capable of growing on different nitroaromatic and nitramine-type explosives [5]. Despite this diversity, only a few microbial enzymes that belong to the nitroreductase and old yellow enzyme (OYE) families have been identified as responsible for catalyzing the breakdown of nitroaromatics, including TNT. The chemical structure of this compound makes it particularly difficult to transform, due to the symmetrical arrangement of the three electron-withdrawing nitro groups, which create steric hindrance and prevent the conventional electrophilic attack by oxygenase enzymes (which is not the case for mono- and dinitrotoluenes) [12]. On the other hand, depending on the oxygen requirements of bacteria, the transformation can be carried out either aerobically or anaerobically, leading to multiple reduction steps or complete reduction, respectively. Under aerobic conditions, bacterial TNT transformation generally occurs via reduction of nitro groups and/or aromatic ring reduction [13]. The main enzymes involved in these reactions are xenobiotic reductases (XENr) [14,15,16], *N*-ethylmaleimide reductases (NEMr) [17,18] and pentaerythritol tetranitrate reductases (PETNr) [19,20] of the OYE family, and nitroreductases type I of the nitroreductase family [21]. Unlike OYEs, nitroreductases act only by reducing nitro groups of TNT and do not have the ability to break down the aromatic ring.

Among the main bacteria capable of carrying out TNT transformation are members of the genus *Pseudomonas*. Different species, such as *P. putida*, *P. fluorescens*, and *P. aeruginosa*, have been reported to have enzymes that are capable of metabolizing TNT [14,22,23]. Members of this genus are Gram-negative and rod-shaped bacteria with great metabolic versatility that allows them to thrive under harsh environmental conditions, including those from extreme geographical locations, such as Antarctica [24,25]. This remote continent harbors sites with unique geology and microbiological diversity, such as Deception Island, a still poorly explored ecosystem that, in recent years, has become a highly interesting hotspot for the search and discovery of novel microorganisms and enzymes with potential biotechnological applications [26,27,28].

In this context, in a previous study, we reported the isolation of three Antarctic *Pseudomonas* (named TNT3, TNT11, and TNT19) and the assessment of their TNT-transforming activity [29]. Notably, the three isolates showed higher consumption and growth on TNT than *P. putida* KT2440, one of the best-studied TNT-transforming bacteria [22,30]. In light of those results, and in order to gain a deeper understanding of the biotransforming capability of our isolates, we decided to investigate the genomic features that could explain the observed TNT-transforming phenotype, using an in-depth genome-wide analysis and comparative genomics approach. To do this, we started by selecting 21 pseudomonads genomes according to 5 criteria. Firstly, we searched for species with experimentally demonstrated TNT-transforming activity, aiming to identify genomic elements both shared and unique to our isolates that might be involved in TNT transformation. The same criterion was used to search for species capable of degrading other aromatic xenobiotics. Then, we searched for species that were isolated exclusively from Antarctica, aiming to determine whether TNT-degrading genes are a common feature among them, or instead, a particularity of our isolates. Additional criteria based on phylogenomic and phylogenetic closeness were used for selecting other species that could help us assess the genetic novelty of our isolates and determine whether they correspond to novel species. Lastly, to evaluate the potential use of our isolates as bioremediation agents, pathogenic species were selected to compare their pathogenic profile with that of our isolates.

The comparative genomic analyses carried out in the present study, consisting of comprehensive functional annotation, metabolic potential analysis, pangenome reconstruction, and homology search for TNT metabolism-related enzymes, revealed that our isolates exhibit unique metabolic potential and also a noteworthy potential to degrade TNT and other aromatic xenobiotics, such as fluorobenzoate, xylene, and bisphenol A. Furthermore, most in-depth analyses revealed that our isolates have unique enzymes that are involved in central metabolic routes, such as the β-ketoadipate pathway (where several aromatics degradation routes converge) and also in peripheral metabolic routes (for direct TNT degradation) that are not present in other well-known TNT-transforming pseudomonads. Additionally, the phylogenetic/phylogenomic analyses and pathogenicity-related genes search indicate that our isolates are novel non-pathogenic *Pseudomonas* species. To the best of our knowledge, this is the first comparative genomics report on TNT transformation by *Pseudomonas* species.

## 2. Materials and Methods

### 2.1. Bacterial Species and Culture Conditions

The bacterial isolates *Pseudomonas* sp. TNT3, TNT11, and TNT19 used in this work (from now on referred as ‘TNT isolates’) were previously isolated from soil samples collected from Deception Island, Antarctica, during the 54th Chilean Antarctic Scientific Expedition in 2018. The isolates were cultured for DNA extraction as previously reported [29].

### 2.2. Public Data Acquisition

In this study, the genomes of TNT isolates were compared with those of 21 other pseudomonads, which were selected and classified into 5 groups according to the following criteria: *Group 1*. *Pseudomonas* spp. with experimentally demonstrated TNT transformation capability. To the best of our knowledge, only two *Pseudomonas* strains meet this criterion, *P. putida* JLR11 and *P. putida* KT2440. Unfortunately, the genomes of other reported TNT-transforming pseudomonads are not publicly available [2,31,32]; *Group 2*. *Pseudomonas* spp. with experimentally demonstrated xenobiotics degradation capability, including aromatics other than TNT, *P. aeruginosa* PFL-P1, *P. frederiksbergensis* AS1, *P. veronii* 1YdBTEX2, and *P. veronii* Pvy. These species were selected to evaluate and compare their functional potential with that of the TNT isolates to degrade other aromatic xenobiotics, considering the capability of the former to degrade compounds that are structurally similar to TNT (e.g., benzene, toluene); *Group 3*. *Pseudomonas* spp. from the same geographical origin (Antarctica), *Pseudomonas* sp. GC01, *Pseudomonas* sp. MPC6, *P. deceptionensis* LMG 25555, *P. antarctica* PAMC 27494, *P. fildesensis* KG01, and *P. extremaustralis* 14-3b. These species were selected aiming to investigate whether TNT-transforming capability is a ubiquitous trait among Antarctic isolates or whether it is present only in TNT isolates; *Group 4*. *Pseudomonas* spp. phylogenetically close to TNT isolates, *P. mandelii* PD30, *P. mandelii* DSM 17967, *P. frederiksbergensis* ERDD5:01, *P. frederiksbergensis* AS1, *P. fluorescens* F113, *P. fluorescens* ATCC 13525, *P. veronii* Pvy, and *P. veronii* 1YdBTEX2. These species were used to determine whether our isolates potentially correspond to novel species of the genus *Pseudomonas*. The search and selection of these species were performed based on 16S gene identity and genome distance estimation computed by the “similar genome finder” tool of the PathoSystems Resource Integration Center (PATRIC) server [33]. Additionally, their functional potential to metabolize TNT and other xenobiotics was compared with that of TNT isolates; *Group 5*. Pathogenic *Pseudomonas* spp. Since pathogenic bacteria are not suitable for bioremediation purposes, we included pathogenic species in the analysis in order to evaluate the pathogenicity of our isolates toward humans and plants. Human-pathogenic *Pseudomonas* spp include *P. aeruginosa* PAO1, *P. aeruginosa* UCBPP-PA14. Phytopathogenic *Pseudomonas* spp include *P. syringae* pv. *syringae* strain B301D and *P. syringae* pv. *syringae* strain HS191. The latest available version of each of the genome assemblies mentioned above was considered in the analyses. NCBI’s RefSeq accession codes and details about these genomes are shown in Table S1.

### 2.3. Genome Sequencing and Assembly

High-quality genomic DNA was extracted from TNT isolates using the Wizard Genomic DNA Purification Kit (Promega, Madison, WI, USA), according to the manufacturer’s instructions. The whole-genome sequencing library was prepared from 1 ng of genomic DNA from each isolate using the Illumina Nextera XT kit. DNA library sequencing was conducted on an Illumina MiSeq platform, using 2 × 300 bp paired-end sequencing mode at the Center of Plant Biotechnology (Universidad Andrés Bello, Santiago, Chile). Raw sequencing reads were quality checked using FastQC v0.11.9 [34] and then trimmed and filtered using BBDuk v38.84 [35]. The quality trimmed reads were *de novo* assembled with SPAdes v3.13.1 [36] using the “careful” mode. Contigs of less than 1 kbp were filtered out. Assembly polishing was performed using Pilon v1.24 [37]. Assembly and quality assessment metrics were obtained using QUAST v5.0.2 [38] and coverage statistics were calculated by mapping reads back to the assembled contigs with BBMap v38.84 [35]. The completeness of the draft assemblies was assessed by searching for universal single-copy marker genes and orthologs using CheckM [39] and BUSCO [40] programs, respectively. For BUSCO, the bacteria_odb10 dataset (124 single-copy orthologs) was used, while for CheckM, *Pseudomonas* lineage-specific markers were considered. The draft genome sequences of *Pseudomonas* sp. TNT3, TNT11, and TNT19 have been deposited in the GenBank database (NCBI) under the accession numbers WFGV00000000, JAKNRV000000000, and JAKNRW000000000, respectively.

### 2.4. Genome Annotation and Metabolic Potential Analysis

The *de novo* genome assemblies were annotated using RASTtk [41] implementation available on PATRIC server v3.6.12 [33], PROKKA v1.13 [42] and eggNOG-mapper [43,44]. Comparative metabolic potential plots were generated using the recently published functional annotation pipeline MicrobeAnnotator [45]. Briefly, this latter tool performs a functional annotation of the predicted coding sequences from multiple query genomes, by applying an iterative search against four different databases (KOfam, Swiss-Prot, TrEMBL, and RefSeq), aiming to obtain a KEGG Orthology identifier (KO) for each protein. When the iterative search is complete, the identifiers associated with all proteins in each genome are extracted, and the metabolic potential is calculated based on the total steps in a module, the proteins (KOs) required for each step, and the KOs present in each genome. The output is a matrix of KEGG metabolic modules completeness, which is visualized through a bar plot and a heat map, showing module completeness per genome. In this study, RASTtk gene predictions were used as input for MicrobeAnnotator.

To obtain comparative visualizations of the annotation results, we generated heat maps using the “heatmap.2” R package and subjected them to multivariate statistical analyses using the hierarchical clustering method, as implemented in the *hclust* R function. Briefly, this function performs a hierarchical cluster analysis using a set of dissimilarities (computed using squared Euclidean distances) for the *n* objects being clustered (complete agglomeration method was used for clustering). More details on this function are available at https://www.rdocumentation.org/packages/stats/versions/3.6.2/topics/hclust, accessed on 24 July 2022.

### 2.5. Pangenome Analysis

Pangenome reconstruction was performed using Roary v3.12 [46] using default parameters. Annotated assemblies in the gff3 format produced PATRIC/RASTtk and were modified using in-house scripts to meet the specific formatting accepted by Roary as the input for pangenome calculations, as described in https://github.com/The-Sequence-Ontology/Specifications/blob/master/gff3.md, accessed on 12 January 2022. Downstream analysis followed the guidelines in https://sanger-pathogens.github.io/Roary/, accessed on 12 January 2022. Roary’s core- and pangenomes were inferred by applying 95% blastp identity threshold for clustering protein sequences. Core genes were defined as those occurring in ≥99% of genomes (hard-core) and in the range 95–99% (soft-core); accessory genes were defined as those occurring in 15–95% (shell) and ≤15% (cloud) of the genomes; unique genes were defined by Roary as those specific to a single genome or set of genomes according to an identity threshold of 95%. Post-processing of pangenome data was performed using in-house scripts. Pangenome was queried using Roary’s *query_pan_genome* script to establish the gene differences between different sets of species and to identify unique genes.

### 2.6. Phylogenetic and Phylogenomic Analyses

To infer phylogenetic relationships among the 24 pseudomonads considered in this study, the core genes deduced by Roary were used to conduct a multilocus sequence analysis (MLSA). To do this, core genes were extracted from each genome, aligned with MUSCLE [47] and trimmed using BMGE [48] with the DNAPAM100 matrix. Trimmed alignments were then concatenated with the ‘concat’ command from SeqKit toolkit [49]. The resulting concatenated alignment was used to infer a maximum likelihood (ML) tree by conducting 20 ML tree searches under the standard GTRGAMMA model with randomized accelerated maximum likelihood (RAxML) v8.2.12 [50]. Support values for the best scoring tree were computed using the bootstrap procedure considering 100 replicates. The phylogenetic tree was visualized using iTOL v6 [51]. For phylogenomic analysis, pairwise average nucleotide identities (ANI) between TNT isolates and the other pseudomonads were calculated using the PyANI v0.2.11 python module [52] based on BLAST+ (ANIb) and MUMmer (ANIm) algorithms. The results were visualized with the heatmap.2 R package [53].

### 2.7. Prediction of Mobile Genetic Elements and Other Specific Genomic Features

Mobile genetic elements (MGEs), such as prophages, genomic islands (GIs), insertion sequences (ISs), clustered regularly interspaced short palindromic repeats (CRISPR) and their associated (Cas) proteins, were predicted in the genomes of all TNT-transforming species. PHASTER [54] was used to identify putative prophage regions. GIs were predicted with IslandViewer4 using reference genomes [55]. Isolates TNT3 and TNT19 were compared with *P. mandelii* JR-1, TNT11 was compared with *P. veronii* Pvy and strains KT2440 and JLR11 were compared with *P. putida* KT2440. ISs were identified using the ISEScan analysis pipeline [56]. CRISPR-Cas sequences were predicted using CRISPRCasFinder [57]. Virulence factors and antibiotic resistance genes were identified in the genome annotation generated by PATRIC [33] using the Virulence Factor Database (VFDB) [58] and the Comprehensive Antibiotic Resistance Database (CARD) [59], respectively. In addition, the prediction of secondary metabolite production gene clusters was performed using the antiSMASH 6.0 tool [60].

### 2.8. Identification and Analysis of Putative TNT Metabolism-Related Enzymes

To identify enzymes directly involved in TNT-degradation (i.e., direct conversion of TNT molecule into metabolites), such as nitroreductases, OYEs, and azoreductases (from now on, collectively referred to as TNT-degrading enzymes), in the genomes of TNT isolates, we initially inspected the RASTtk automatic annotation results. Then, we conducted a comprehensive homology search against a set of experimentally characterized TNT-degrading enzymes. Gene products annotated with non-specific names by RASTtk (e.g., NADH-dependent oxidoreductase) were compared against the UniProt Knowledgebase database (UniProtKB) [61] for manual curation. For specific homology searches, all translated CDSs previously predicted for the genomes of TNT isolates were queried (blastp) against a local database of characterized TNT-degrading enzymes from *Pseudomonas* spp. retrieved from GenBank, including nitroreductases PnrA (AAM95986.1) and PnrB (AAM95987.1) from *P. putida* JLR11; OYEs XenA from *P. putida* II-B (AAF02538.1) and *P. putida* KT2440 (AAN66878.1), XenB from *P. fluorescens* I-C (AAF02539.1) and *P. putida* KT2440 (AAN66545.1), XenC (AAN68098.1), XenE (AAN67100.1), and NEMr (AAN68781.1) from *P. putida* KT2440. In the same way, MexE, MexF, and OprN subunits of the MexEF-OprN efflux pump were also searched, owing to their role in xenobiotic extrusion. Then, the sequences found were characterized by identifying the conserved residues described for each type of enzyme by performing multiple sequence alignments (MSA) with MUSCLE [47]. Additionally, the theoretical molecular mass of each sequence was calculated with the ExPASy’s ProtParam tool [62]. All the above-described analyses were also performed on *P. putida* strains JLR11 and KT2440 genomes for comparative purposes. Additionally, a neighbor-joining cladogram was constructed for nitroreductase, xenobiotic reductase, and azoreductase enzymes using the Jones–Taylor–Thornton (JTT) substitution model and 100 bootstrap replicates, using MAFFT server [63].

### 2.9. Reconstruction of TNT Metabolic Pathways

Since many of the metabolic pathways for nitroaromatic degradation are poorly characterized (or not characterized at all), particularly those for TNT detoxification, we compiled the information available in the literature regarding TNT catabolism to propose a tentative diagram of the reaction routes where *Pseudomonas* TNT metabolism-related enzymes participate. The putative TNT-degrading enzymes found in all TNT-transforming species analyzed in this study were mapped to the routes where they probably participate according to their functional annotations and/or experimental evidence.

## 3. Results and Discussion

### 3.1. General Genomic Features of TNT Isolates

Shotgun DNA sequencing yielded 5.6, 3.2, and 4.8 million reads, with Q ≥ 30 mean sequence quality for isolates TNT3, TNT11, and TNT19, respectively. After reads pre-processing, the mean sequence quality was Q ≥ 35. The *de novo* assembly of filtered reads and polishing steps generated assemblies of 138, 718, and 92 contigs with N50 values of 93,061, 13,749 and 166,334 bp for TNT3, TNT11, and TNT19, respectively (Table 1). The draft genome size and G+C content of TNT3 and TNT19 were similar, both being around 6.46 Mbp and 58.6% G+C (Figure S1). In contrast, the genome size of TNT11 was smaller (5.86 Mbp) and the G+C content slightly higher (60.44%). Nevertheless, the genome size and G+C content of the three isolates were in agreement with those reported for other pseudomonads [24]. High sequencing depth was obtained for each sample, being the mean sequence coverage 110×, 174×, and 151× for TNT3, TNT11, and TNT19 genomes, respectively. Draft genome completeness estimations by BUSCO and CheckM were congruent, revealing that genomes of TNT3 and TNT19 are virtually complete (~100%), whilst the genome of TNT11 is close-to-complete (96–97%) (Figure S2a). No missing BUSCOs were found in TNT3 and TNT19 and only one single-copy ortholog was missing in the TNT11 genome. In addition, the metrics estimated by CheckM revealed minimal levels of contamination (0.45–2%) (Figure S2b).

### 3.2. Annotation and Metabolic Potential Analysis

Draft genome assemblies of the TNT isolates were initially annotated with PATRIC/RASTtk. As a result, the total number of coding sequences (CDSs) in these genomes ranged between 6120 and 6322, of which nearly 1400 (22%) were annotated as hypothetical proteins. In addition, around 5 rRNA-encoding genes and 59 tRNA-encoding genes were predicted in the 3 genomes. A summary of the general features of the assembled genomes and their annotation is shown in Table 1. Then, the predicted CDSs were classified into “clusters of orthologous groups of proteins” (COGs) functional categories by mapping them to the eggNOG database (v5.0) with eggNOG-mapper. As shown in Figure S3, the most abundant COGs are mainly involved in central metabolism and energy production. The top three most enriched COG categories were S (function unknown), E (amino acid transport and metabolism) and K (transcription), comprising 18, 8.9 and 8.8% of all proteins classified, respectively. Additionally, about 6% of the proteins in TNT isolates could not be classified in any functional category (“not classified” in Figure S3). Although COG abundance profiles among these isolates are quite similar, it is possible to observe the enrichment of categories such as P (inorganic ion transport and metabolism) in TNT3, N (cell motility) in TNT11, and L (replication, recombination and repair) in TNT19, which are consistent with the RASTtk annotation results and hint at distinctive metabolisms.

In order to compare the overall metabolic potential encoded by TNT isolates with that of the other 21 selected pseudomonads, a comprehensive annotation was carried out using the MicrobeAnnotator pipeline. A matrix that compiled the predicted completeness of all KEGG modules per genome was generated and graphically summarized as a bar plot and a heat map (considering only metabolic modules at least 80% complete in at least one of the compared genomes). The MicrobeAnnotator results revealed that genomes of the TNT isolates and *P. veronii* Pvy have noticeably fewer complete KEGG metabolic modules (<50) than the remaining genomes, with TNT3 having the fewest complete modules, as shown in the bar plot in Figure S4. It was also observed that TNT3 and TNT19 share a higher similarity of metabolic potential (in terms of overall KEGG modules completeness) with each other than with TNT11, as they were clustered together in a separated clade, whilst TNT11 formed a clade together with the highly versatile species *P. veronii* Pvy (Figure S5).

**Figure 1:**
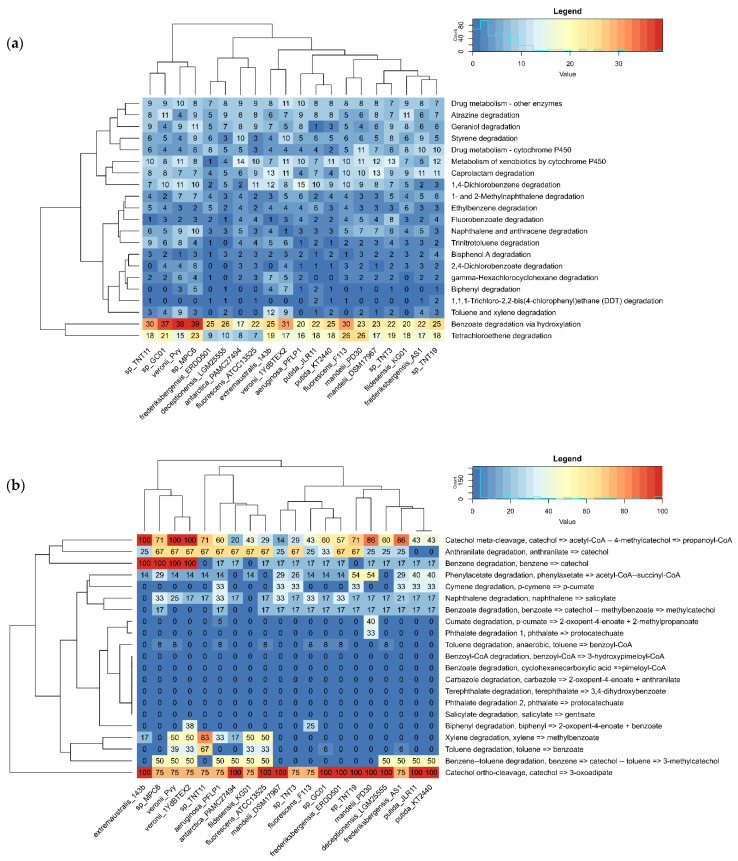
Xenobiotics and aromatics degradation potential of pseudomonads analyzed in this study. (**a**) Count of genes in “xenobiotics degradation and metabolism” pathway according to PATRIC/RASTtk annotation; (**b**) percentage of completeness of “aromatics degradation” KEGG modules obtained with MicrobeAnnotator for TNT isolates and all non-pathogenic pseudomonads included in the analysis.

To explore and compare in more detail the abundance of genes that encode catabolic (degradative) enzymes in the set of genomes, annotation results of the whole PATRIC’s “xenobiotics degradation and metabolism” pathway and MicrobeAnnotator’s “aromatics degradation” KEGG pathway were compiled into matrices of gene counts by category and percentage of completeness by module, respectively. The matrices were represented as heat maps for easy interpretation (Figure 1). Pathogenic species (Group 5) were not included in this analysis, due to their low functional potential to degrade xenobiotics.

According to the RASTtk annotation, all the analyzed species have the potential to degrade xenobiotics to a different extent. Nevertheless, the catabolic potential seems to be enriched in a cluster comprising four species (TNT11, *Pseudomonas* sp. GC01, *P. veronii* Pvy, and *Pseudomonas* sp. MPC6), which features the largest number of enzymes for “benzoate degradation via hydroxylation”, as shown in Figure 1a. It is worth mentioning that all of these species, except for *P. veronii* Pvy, are of Antarctic origin. When focusing only on TNT isolates, we observed that TNT3, TNT11, and TNT19 have the highest metabolic potential to degrade fluorobenzoate, TNT, and bisphenol A, respectively. In particular, the number of genes in the “trinitrotoluene degradation” pathway found by PATRIC in these isolates is greater than that of *P. putida* KT2440, which might explain to some extent our previously reported experimental results, where TNT isolates showed a higher TNT transformation rate compared to the KT2440 strain [29]. Notably, among all pseudomonads compared, TNT11 is the one with the highest gene count for the “trinitrotoluene degradation” pathway (Table S2). According to our previous results, it would have been expected that isolate TNT3 would have more enzymes in this pathway, since it showed to be more efficient in degrading TNT than isolate TNT11. Nevertheless, it is evident that not only a larger genetic repertoire is important for catabolic functions but also the conditions that induce gene expression and the catalytic efficiency of the encoded enzymes.

Compared to PATRIC/RASTtk, MicrobeAnnotator provides more exhaustive annotations thanks to its iterative approach using KOfam, Swiss-Prot, TrEMBL, and RefSeq databases, which improves the assignment of functions to unannotated proteins and also allows us to evaluate the level of completeness of the different KEGG metabolic modules in a genome. When analyzing the results for “aromatics degradation” pathway modules with this tool (Figure 1b), it is clear that the degradation potential of the species analyzed is concentrated in catechol degradation via *ortho*-cleavage (KEGG module: M00569), which is over 75% complete in all of them. Interestingly, this module is only complete in TNT19, as well as in *P. putida* strains and some other Antarctic species (*Pseudomonas* sp. GC01 and *P. deceptionensis* LGM25555). On the contrary, catechol degradation via *meta*-cleavage (KEGG module: M00569) is complete only in *P. veronii* strains and *P. extremaustralis* species, although in TNT11 and TNT19, this module is also near complete (71%), unlike the *P. putida* strains. Here, it is worth mentioning that the origin of aromatic compounds degraded by bacteria can be both natural (e.g., amino acids and petroleum-derived hydrocarbons) and anthropogenic (e.g., nitroaromatics, dioxins, polychlorobiphenyls and pesticides). These compounds are generally catabolized by several peripheral metabolic pathways (e.g., toluene, naphthalene, chlorobenzoate, phenylalanine, etc.) that converge in a few central intermediates (e.g., catechol or its derivatives, homogentisate, hydroquinone, etc.), which are then converted into tricarboxylic acids (TCA) cycle intermediates by a small number of common central pathways [64,65]. In the case of catechol, it can be degraded via the *ortho*-cleavage pathway, resulting in succinyl-CoA and acetyl-CoA and via the *meta*-cleavage pathway, resulting in acetaldehyde and pyruvate. All of these final products can enter directly into the TCA cycle [65].

It was also observed that anthranilate degradation (KEGG module: M00637) is widely distributed in the pseudomonads analyzed, although it is not complete in any of them. Interestingly, in TNT isolates, this module is 67% complete, whereas in *P. putida* strains, it is absent. Since anthranilate is a common intermediate formed during the aerobic catabolism of some nitroaromatics, which is then converted to catechol [65], these results may shed light on why TNT isolates are more efficient at transforming TNT than KT2440 [29]. Some other modules, such as “benzene degradation” (KEGG module: M00548), are incomplete or absent in most genomes, including TNT isolates and TNT-transforming *P. putida* strains, being complete only in *P. veronii* strains and in the Antarctic species *P. extremaustralis* 14-3b and *Pseudomonas* sp. MPC6. Another interesting result was that among all pseudomonad genomes analyzed, isolate TNT11 is the species with the most complete xylene module (KEGG module: M00537), which is apparently not a common feature among any of the other pseudomonad genomes analyzed. The same was observed for the toluene degradation module (KEGG module: M00538). Similarly, isolate TNT19 stands out as one of the two species with the most complete phenylacetate degradation module (KEGG module: M00878), which is also interesting, since the degradation of this compound differs from classical mechanisms of aromatics degradation [65] and is not a common feature among the pseudomonads analyzed. Finally, none of the species included in the heat map would be capable of degrading aromatics of complex chemical structures, such as terephthalate, carbazole, and benzoyl-CoA, among others. In summary, the results obtained with these two functional annotation tools revealed particular features in our isolates that highlight their xenobiotic catabolic potential, and at the same time could partly explain their TNT-transforming phenotype.

### 3.3. Pangenome Analysis and Unique Genes

To investigate genomic differences and similarities among the 24 pseudomonads selected for this study, we used Roary to perform a pangenome analysis based on PATRIC/RASTtk-predicted CDSs. Genes were re-annotated using the standalone eggNOG-mapper tool to assign COG and KEGG categories to core and accessory genes. As expected, due to the diversity of the species analyzed, the resulting pangenome was open, as indicated by the increase in cumulative genes with the number of genomes included (Figure S6a) and the small number of core genes (Figure S6b). Of 30,253 total orthologous gene clusters in the pangenome of the 24 species, the core genome comprised only 27 (hard) and 2 (soft) core genes, with the accessory genome containing 4703 shell genes and 25,521 cloud genes, whereas unique genes ranged from 13 (*P. aeruginosa* PAO1) to 354 (*Pseudomonas* sp. GC01) (Figure S6c). The number of unique genes obtained for isolates TNT3, TNT11, and TNT19 was 216, 302, and 262, respectively. Mapping of these genes to the KEGG pathways revealed that they are mainly distributed in “metabolic pathways” (79 genes), “biosynthesis of secondary metabolites” (38 genes), “two-component system” (31), “microbial metabolism in diverse environments” (29 genes) and “ABC transporters” (22 genes). Nevertheless, around 45% of the unique genes had no KEGG Orthology (KO) assignment.

To simplify the analysis and identify differences between the TNT isolates and the other 21 pseudomonads, we separated them into 5 groups, plus an additional group consisting exclusively of TNT isolates genomes, to construct sub-pangenomes (Table S3). As a result, we observed that core genes in the five groups were mainly related to central metabolic functions (translation, transcription, transport, and energy production). The same was observed for the core genes obtained for the sub-pangenome of TNT isolates genomes only, which also was the largest (613 genes), followed by Group 4 (541 genes) and Group 3 (338 genes). Conversely, the smallest core genome was obtained for Group 2 (17 genes) as expected, due to the diversity of species in it. Regarding accessory genes, we observed that most genes were classified as “function unknown” by COG in all sub-pangenomes, being the largest set that of Group 5 (11,806 genes). This result is not unexpected, since accessory genes are usually related to antibiotic resistance and virulence factors acquired via horizontal gene transfer, elements commonly found in *P. aeruginosa* (human pathogen) and *P. syringae* (phytopathogen) strains [24], which are also more phylogenetically distant to TNT isolates than all species in the remaining groups.

We then focused our attention on specific differences between our isolates and those other *Pseudomonas* species with experimentally demonstrated TNT-transforming activity, exclusively (i.e., *P. putida* strains KT2440 and JLR11). For this purpose, we queried the sub-pangenome of Group 1 to identify unique genes in our isolates that are not present in *P. putida* strains. Genes were considered unique if they were below the identity threshold set by Roary calculations, as mentioned in Section 2.5. Then, the annotation of these genes was extracted from the MicrobeAnnotator output and plotted as a heat map for comparative purposes. As a result, 2014, 688, and 2035 unique genes were identified in isolates TNT3, TNT11, and TNT19, respectively. When inspecting the heat map of Figure S7, we observed that, interestingly, among these unique genes are those involved in the “catechol *ortho*-cleavage” route (KEGG module: M00568), which is part of the β-ketoadipate pathway. The enzymes encoded by these genes are listed in Table 2.

The β-ketoadipate pathway is a widely distributed route for the degradation of aromatic compounds in both bacteria and fungi, in which different catabolic pathways of aromatics (including nitroaromatics) converge. It consists of two main parallel *ortho*-cleavage branches, one for catechol degradation and the other for protocatechuate degradation, both of which are converted into other metabolites that can directly enter the TCA cycle, as mentioned above [66,67]. Interestingly, among the unique genes of both TNT3 and TNT19, a gene encoding the β chain of protocatechuate 3,4-dioxygenase (P3,4O), a key ring-opening enzyme in the β-ketoadipate pathway, was found. As demonstrated for the first time by a recent knockout study on a deletion mutant for this gene in *Buttiauxella* sp. S19-1, the P3,4O enzyme is involved in the degradation of TNT and its metabolites, possibly catalyzing downstream reactions in this pathway [11]. It is worth emphasizing that the genes encoding P3,4O in both TNT3 and TNT19 share sequence identity below 50% when compared to that of *P. putida* strains KT2440 and JLR11. Furthermore, two additional genes were also found in these isolates, encoding the xenobiotic reductases XenB and XenE, respectively (Table 2). Both enzymes participate in peripheral routes by directly degrading TNT [14,22]. More details regarding these two enzymes are addressed below (Section 3.7 and Section 3.8). All these results indicate that TNT isolates bear a particular set of enzymes, different enough from that of *P. putida* strains to be considered unique by the pangenome analysis tool used, which could explain their more efficient TNT-transforming phenotypes, as previously observed [29].

### 3.4. Phylogenetic and Phylogenomic Analyses

**Figure 2:**
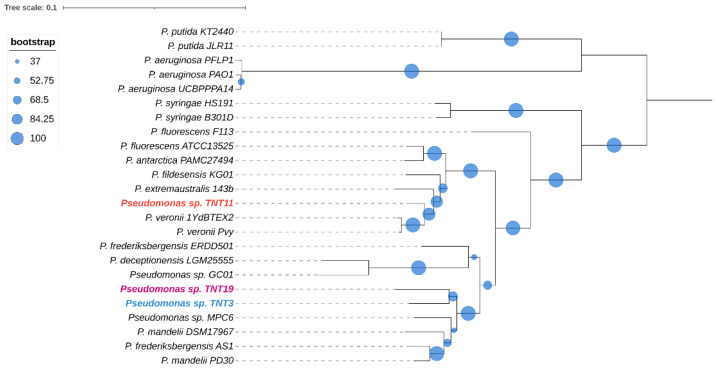
Maximum likelihood phylogenetic tree based on concatenated core gene sequences from the 24 pseudomonads considered in this study. The tree was generated with RAxML using the GTRGAMMA model. The size of the blue circles represents the percentage of bootstrap support for each branch (based on 100 iterations). The tree was midpoint rooted.

To obtain a deeper insight into the phylogenetic relationships between TNT isolates and the other 21 pseudomonads, a core genome multilocus sequence analysis (MLSA) approach was used. The core genome was composed of only 27 genes, which indicates that the species included in the analysis have divergent genomes. The phylogenetic analysis shown in Figure 2 revealed that isolates TNT3 and TNT19 are closely related, sharing a clade along with four other species, which also includes the Antarctic strain *Pseudomonas* sp. MPC6. On the other hand, TNT11 is more closely related to *P. veronii* strains (aromatic xenobiotics degraders) [68,69] than to other Antarctic close neighbors, such as *P. fildesensis* KG01 and *P. extremaustralis* 14-3b.

**Figure 3:**
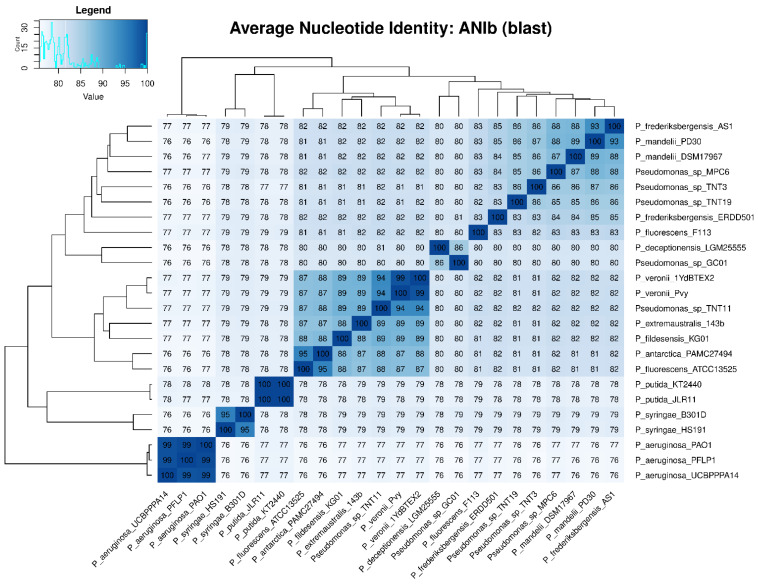
Average nucleotide identity (ANI) heat map based on BLASTn algorithm (ANIb) among the 24 pseudomonads used in this study. ANI values were calculated with PyANI after blastn alignment. Values are shown as percentages (%) of aligned nucleotides. ANI values above 95% between two genomes indicate that they belong to the same species.

To determine the taxonomic classification of the TNT isolates, ANI values were calculated. An ANI value below 95% indicates that two microorganisms are different species [70]. Figure 3 shows the ANIb values of all the 24 pseudomonads used in this study, which were in good agreement with those of ANIm (Figure S8). The highest ANIb values for TNT3 and TNT19 were observed when these isolates were compared to *P. mandelii* PD30 (denitrifying bacterium) [71] and *P. frederiksbergensis* AS1 (aromatic degrader) [72], respectively, both reaching near 86% identity (clearly below the 95% threshold). Regarding TNT11, the highest ANIb value was observed when it was compared to *P. veronii* 1YdBTEX2 (aromatics degrader) [68], reaching nearly 94% identity (very close to the 95% threshold). These results strongly suggest that TNT3 and TNT19 are novel species, whilst TNT11 (given the proximity of its ANI to the species definition value) would require further analyses (genotypic and phenotypic) to be proposed as a new species.

### 3.5. Mobile Genetic Elements (MGEs)

Since MGE-mediated horizontal gene transfer is a major mechanism for the acquisition of novel accessory genes, including those for xenobiotics degradation [73,74], we searched for MGEs, such as prophages, insertion sequences (ISs), genomic islands (GIs), and CRISPR-Cas, in all the TNT-transforming species considered in this study. As a result, putative prophage regions were identified, representing 0.9%, 2.3%, 1.8%, 3.6%, and 3.8% of the total genome size of TNT3, TNT11, TNT19, JLR11, and KT2440, respectively (Table S4). These MGEs have been reported before in other pseudomonads [24,75,76]. However, the low number of prophage regions in TNT isolates (notably in TNT3), as opposed to that observed in the *P. putida* strains, reveals that the former have less foreign genetic material from bacteriophages in their genomes.

In order to investigate whether ISs are flanking TNT-degrading genes, we also conducted a specific search for these elements, since it has been shown that they can modulate the gene expression, including that of xenobiotics-degrading genes [77,78,79,80]. As a result, many complete and partial ISs were identified in the genomes of the TNT-transforming species, being more abundant in *P. putida* strains (42–50 ISs), whereas TNT isolates have less than 40 ISs, especially TNT3, which has only 15. Nevertheless, no ISs flanking TNT-degrading genes were found in any of the genomes analyzed.

Since GIs often carry virulence factors and even entire metabolic pathways for xenobiotics degradation that can be acquired via horizontal gene transfer (HGT) [81,82], we also searched for them in TNT-transforming species. The results revealed the presence of GIs in all of them, indicating that HGT events occurred in their genomes (Figure S9). The number of GIs predicted for TNT3, TNT11, TNT19, JLR11, and KT2440 was 43, 24, 52, 32, and 69, respectively. The high GI content in KT2440 evidences its high genomic plasticity, compared with the other species analyzed. It is worth mentioning that the vast majority of genes within the GIs in the genome of TNT-transforming species encode hypothetical proteins and phage-related proteins, which is consistent with other pseudomonad genomes [83,84,85]. Interestingly, a GI sequence containing a prophage region and a putative gene cluster encoding three TNT-degrading enzymes (two xenobiotic reductases and one nitroreductase) was found in JLR11 (Figure S9d) and KT2440 (Figure S9e) (this finding is addressed in more detail in Section 3.7), suggesting that these catabolic genes could have been acquired via horizontal gene transfer in both strains. Conversely, genes encoding putative TNT-degrading enzymes in TNT isolates were not contained within GIs, which indicates that these genes could have been vertically inherited. Lastly, no CRISPR-Cas sequences were found in any of the TNT-transforming species.

Since Antarctica is still one of the most isolated and pristine continents on Earth (particularly some sites, such as Deception Island), the indigenous bacteria that inhabit it are less exposed to horizontal gene transfer events mediated by non-indigenous microorganisms, which would explain, to some extent, the low content of MGEs in TNT isolates.

### 3.6. Pathogenic Profile and Other Specific Features of TNT Isolates

**Figure 4:**
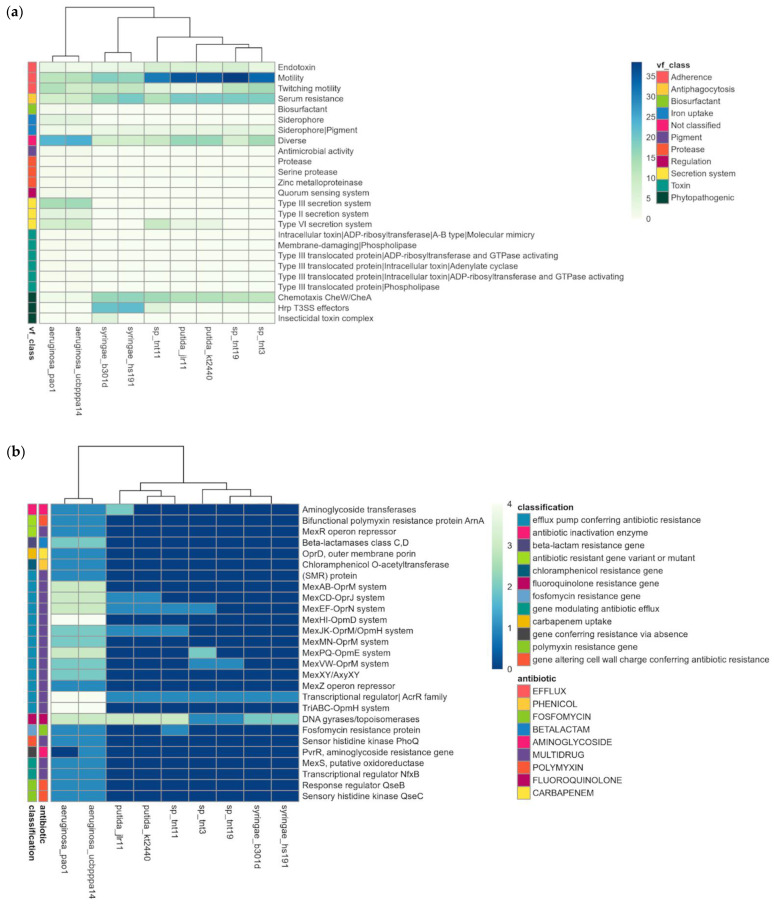
Pathogenic profile of TNT isolates as compared with other pathogenic and non-pathogenic pseudomonads. (**a**) Heat map of putative virulence factors; (**b**) heat map of putative antibiotic resistance genes.

To assess the pathogenicity of TNT isolates, we searched for putative virulence-related and antibiotic resistance genes in their genomes. For this, they were compared with human pathogenic (*P. aeruginosa* strains PAO1 and UCBPP-PA14) [86,87] and phytopathogenic (*P. syringae* pv. *syringae* strains B301D and HS191) [88] species. *P. putida* strains JLR11 and KT2440 were also included in the analysis. The results revealed that TNT isolates have few key virulence factors to infect humans and animals (e.g., production of siderophores, type III and IV secretion systems) and plants (e.g., chemotaxis, Hrp T3SS effectors) (Figure 4a) [89]. Indeed, TNT isolates showed a virulence profile very similar to that of the non-pathogenic bacterium KT2440 [24,90]. The same was observed for antibiotic resistance (Figure 4b), as only a few genes were found in TNT isolates, mainly those encoding multidrug efflux pumps, such as Mex-type, which are not only involved in the extrusion of antibiotics but also in the detoxification of other xenobiotics [30,91]. In fact, the induction of the genes encoding MexEF/OprN multidrug efflux pumps has been reported for strain KT2440, when grown in the presence of TNT [30].

Since one of our objectives was to evaluate the potential use of TNT isolates for on-site bioremediation strategies, the way they interact with the surrounding indigenous microorganisms through secondary metabolites is relevant [92]. Therefore, we investigated the capability of TNT isolates to synthesize these molecules. As a result, only one putative gene cluster was found in TNT19, which showed 100% similarity with a siderophore gene cluster from *Xanthomonas oryzae* pv. *oryzae* KACC 10,331 involved in xanthoferrin biosynthesis (Figure S10). Xanthoferrin is a virulence-related molecule produced by xanthomonads for growing under low-iron conditions [93]. However, as shown in Figure 4a, the count of siderophore virulence factors in TNT19 was negligible. A straightforward interpretation of these results would imply that TNT isolates are not pathogenic for humans, plants, and other micro/organisms. Hence, they could be safely used as on-site bioremediation agents.

### 3.7. Analysis of Enzymes Involved in TNT Metabolism in TNT-Transforming Species

In general, TNT metabolism-related gene products are annotated by RASTtk with generic names (e.g., nitroreductases and OYEs), which made it difficult to determine their exact correspondence with the already characterized and manually curated TNT-degrading enzymes described elsewhere. For this reason, we constructed a local blast database using these sequences (when available, Section 2.8), in order to perform a homology search using all the predicted CDSs of TNT-transforming species as queries. Additionally, we performed multiple sequence alignments (MSA) to identify conserved residues of each type of enzyme. The results obtained are discussed in the following subsections.

#### 3.7.1. Nitroreductases

Nitroreductases are a group of NAD(P)H-dependent flavoenzymes that are capable of degrading natural nitroaromatic compounds (e.g., chloramphenicol, nitroglycosides), but they can also act on some aromatic xenobiotics that contain nitro groups, such as the explosives TNT and 2,4,6-trinitrophenol. However, this does not imply that all nitroreductases are capable of degrading TNT. In fact, only a few of these enzymes with activity toward this compound have been reported [94], such as the nitroreductases NfsA and NfsB from *Escherichia coli* K-12 [18] and PnrA and PnrB from *P. putida* JLR11 [23]. The sequences of the latter two enzymes were included in the local database built for homology search (Section 2.8). When querying this database using the predicted CDSs of TNT isolates, only low sequence similarities were found. Nevertheless, two of these sequences initially annotated by RASTtk as “nitroreductases” (referred to as NitroR4 and NitroR5 from now on) were found to share 82–94% identity with “putative nitroreductase” enzymes from other pseudomonads available in the UniProtKB database (Table S5, Figure S11a,b). The cladogram in Figure S14a indicates that putative nitroreductases, such as NitroR4, are related to the nitroreductase YdjA (type I, Group B2 nitroreductases) from *E. coli* (UniProt accession: P0ACY1) and *Salmonella enterica* (UniProt accession: A0A379WBF2), whilst NitroR5 is more similar to nitrobenzene reductase nbzA from *P. putida* (UniProt accession: Q9AH39) and *P. oleovorans* (UniProt accession: Q6DLR9). An additional nitroreductase annotated by RASTtk as “oxygen-insensitive NADPH nitroreductase”, exclusive to TNT11 (which we referred to as NitroR6), was found to share 83% identity with a “nitroreductase” from *Pseudomonas mucidolens* in the UniProtKB database (Figure S11c). This enzyme appears to be related to the type I nitroreductases of Group B1 (PnrB and NfsB), as shown in Figure S14a. Since we did not obtain more specific information regarding the classification of any of these three enzymes than that provided by RASTtk, we searched for conserved residues (according to the relative positions predicted by UniProtKB) using MSA. As a result, we found that only NitroR4 has a set of conserved residues for FMN binding.

Regarding *P. putida* strains, in addition to the expected matches with the nitroreductases PnrA and PnrB of strain JLR11 (which were included in the local database) and their respective homologs in KT2440, three other nitroreductase sequences (which we referred to as NitroR1, NitroR2, and NitroR3) annotated by RASTtk did not match the database. Although these sequences are present in the annotation of the two strains (available in GenBank), there is no reported experimental evidence linking them to TNT degradation. However, when performing MSA, we observed that the same conserved residues for FMN binding previously found in the NitroR4 sequence of each TNT isolate were also found in NitroR2 of *P. putida* strains. Moreover, since these sequences share around 70% identity, they are likely to be homologous.

#### 3.7.2. Xenobiotic Reductases

As is the case for nitroreductases, xenobiotic reductases are flavoenzymes that catalyze NAD(P)H-dependent reduction of a broad range of xenobiotic compounds. According to the homology and phylogenetic analysis, enzymes initially annotated by RASTtk as “NADH-flavin oxidoreductases” in TNT isolates were found to correspond to xenobiotic reductases. In each isolate, one XenB-like and one XenE-like enzyme were found, sharing around 93% and 85% identity with XenB and XenE from *P. putida* strains [22,95], respectively (Table S5). Notably, the XenB-like enzyme has the set of conserved residues involved in substrate and cofactors (FMN and NAD(P)H) binding [14], as shown in Figure S12b, while the XenE-like enzyme has only some of those conserved residues (Figure S12d). It is worth mentioning that the XenB-like and XenE-like enzymes in isolates TNT3 and TNT19 were previously found to be encoded by unique genes (Section 3.3). In addition, two additional putative xenobiotic reductases XenA-like and XenC-like were found exclusively in TNT11, which would be homologous to XenA (UniProt accession: Q3ZDM6) and XenC (UniProt accession: Q88K07) from *P. putida* strains [14,22,96], sharing 92% and 73% identity with them, respectively, and forming their own clades in the cladogram of Figure S14b. Noticeably, only the XenA-like enzyme has all the elsewhere described conserved residues involved in substrate and cofactor binding [96,97,98] (Figure S12a), whereas the XenC-like enzyme has only some of them (Figure S12c).

**Figure 5:**
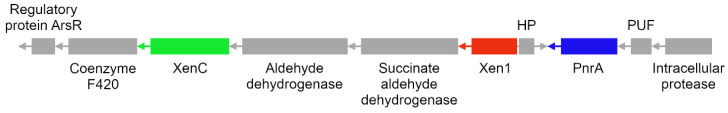
Putative gene cluster encoding TNT-degrading enzymes in *P. putida* strains JLR11 and KT2440. Xenobiotic reductase C (XenC), xenobiotic reductase 1 (Xen1), hypothetical protein (HP), nitroreductase PnrA (PnrA) and protein of unknown function (PUF). 2,4,6-trinitrotoluene (TNT).

Regarding *P. putida* strains JLR11 and KT2440, MSA revealed that an enzyme annotated by RASTtk as “putative xenobiotic reductase” (which we referred to as Xen1) has conserved residues predicted to be involved in cofactor and substrate binding that have been described for xenobiotic reductases in other pseudomonads [96,97,98]. Upon inspecting the genomic context of the gene encoding Xen1, it was found to be part of a putative gene cluster (as mentioned in Section 3.5), along with the already characterized TNT-degrading enzymes XenC and PnrA [22,23], as shown in Figure 5. We also found that this gene cluster encodes the protein ArsR (transcriptional regulator of genes involved in arsenic-driven oxidative stress response), which has been previously found to be positioned near the *xenA* gene in *P. putida* II-B [14]. This is interesting since, as is the case with arsenic, TNT degradation generates oxidative stress in bacteria [99]; for this reason, we think that the regulatory protein ArsR may also be involved in this process. Another gene in this putative cluster encodes the coenzyme F_420_, which can reduce the aromatic ring of TNT but to a much lesser extent than OYEs [100]. It is worth mentioning that bacterial genes encoding xenobiotic-degrading enzymes are usually arranged into gene clusters, such as that described for 2,4,6-trinitrophenol, a nitroaromatic explosive whose chemical structure is highly similar to TNT [101,102,103]. This suggests that *P. putida* strains JLR11 and KT2440 would have a TNT-degrading gene cluster. To the best of our knowledge, this type of cluster has not been described as such in bacteria so far.

#### 3.7.3. Azoreductases

In addition to the already mentioned TNT-degrading enzymes, azoreductases (AzoRs) can also participate in TNT degradation. Generally, AzoRs catalyze the cleavage of azo bonds (–N=N–) of aromatic azo dyes, but some of them have been reported to also have nitroreductase activity, including TNT reduction [104,105,106]. In addition, it has been demonstrated that the gene encoding AzoR1 in *P. putida* KT2440 is upregulated when this bacterium is grown in the presence of TNT [30]. When searching for these enzymes in the analyzed TNT-transforming bacteria, two putative AzoRs (which we referred to as AzoR-a and AzoR-b) were found in all of them. The two AzoRs of each TNT isolate share around 73% and 83% identity with those of strains JLR11 and KT2440, respectively (Table S5). Additionally, these enzymes have the conserved residues involved in substrate and cofactor (FMN and NAD(P)H) binding, as described elsewhere [107] (Figure S13a,b). In addition, one extra AzoR (which we referred to as AzoR-c) was found exclusively in isolate TNT3, sharing 72% identity with AzoR1 from *P. aeruginosa* PAO1 (Table S5) and forming a separated clade, as shown in the phylogenetic cladogram of Figure S14c. Moreover, this enzyme has the conserved residues involved in FMN binding, as reported elsewhere [108] (Figure S13c).

#### 3.7.4. Multidrug Efflux Pumps

Another type of protein associated with TNT metabolism is the multidrug efflux pump MexEF/OprN, which could extrude this compound, as reported for KT2440 [30]. Upon searching the three genes encoding the subunits that compose these kinds of pumps (*mexE*, *mexF*, and *oprN*) in the genomes of TNT isolates and *P. putida* strains, we found copies in all of them. Subunits MexE, MexF, and OprN found in TNT isolates share around 80%, 90%, and 76% identity with those from *P. putida* strains, respectively.

Taken together, all these results confirm that TNT isolates have a set of putative enzymes that are theoretically capable of transforming TNT. Furthermore, considering the good performance of these isolates in transforming this compound in experimental trials [29], we hypothesize that differences at the amino acid level found between their enzymes and those of the *P. putida* strains might be contributing to their affinity toward TNT, which would make them more efficient. To test this hypothesis, further studies including both theoretical (e.g., docking and molecular dynamics) and experimental (e.g., enzyme activity assays and site-directed mutagenesis studies) analyses are required.

### 3.8. Reconstruction of TNT Degradation Pathways

The understanding of the molecular mechanisms that underlie xenobiotic degradation remains a major challenge. Unraveling these mechanisms and genetic adaptations that lead to the evolution of metabolic pathways that allow microorganisms to survive on complex compounds is critical for the development of effective bioremediation strategies. In general terms, the known molecular mechanisms for xenobiotic degradation include recognition and uptake, activation of catabolic genes/enzymes, and extrusion [109,110]. The initial recognition and uptake of these compounds occurs both via passive diffusion or active transport (e.g., major facilitator superfamily transporters, outer membrane porins), which are part of the molecular stress response mechanisms of bacteria [65,111,112]. Once inside the cell, catabolic genes are induced and xenobiotics undergo transformation by peripheral enzymes (e.g., monooxygenases, dioxygenases, nitroreductases, laccases, dehalogenases, peroxidases, etc.) that convert these compounds into a limited number of less toxic derivatives, such as the central metabolic intermediates protocatechuate and catechol [109,113]. As mentioned above, these intermediates act as substrates for enzymes that cleave the aromatic ring. When xenobiotics are not metabolized, they can also be directly extruded by efflux pumps (e.g., multidrug and toxic compound extrusion (MATE) family, Mex-type multidrug resistance efflux pumps, ATP-binding cassette transporters, etc.) as a defense mechanism that prevents cytotoxic concentrations from being reached [65,111].

**Figure 6:**
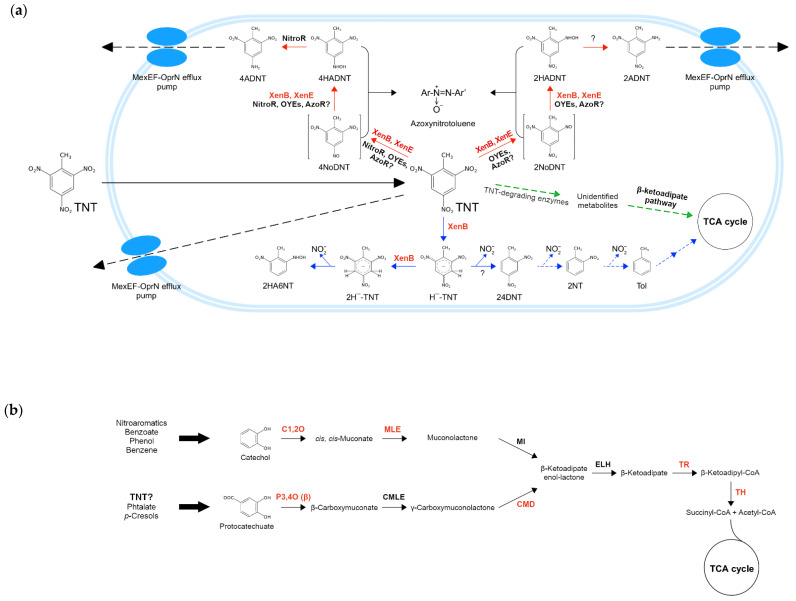
Schematic overview of the main TNT degradation pathways in *Pseudomonas* spp. under aerobic conditions. (**a**) The peripheral pathways nitroreduction and denitration are shown with red and blue arrows, respectively. Furthermore, the central pathway β-ketoadipate is shown with green arrows. Discontinuous arrows indicate the proposed steps. TNT-degrading enzymes are as follows: xenobiotic reductase B (XenB), xenobiotic reductase E (XenE), azoreductase potentially involved (AzoR?), nitroreductase (NitroR), old yellow enzymes (OYEs), and unidentified enzyme (?). Unique enzymes of TNT isolates are shown in red letters. 2,4,6-Trinitrotoluene (TNT), 4-nitroso-2,6-dinitrotoluene (4NoDNT), 4-hydroxylamino-2,6-dinitrotoluene (4HADNT), 4-amino-2,6-dinitrotoluene (4ADNT), 2-nitroso-4,6-dinitrotoluene (2NoDNT), 2-hydroxylamino-4,6-dinitrotoluene (2HADNT), 2-amino-4,6-dinitrotoluene (2ADNT), hydride-Meisenheimer complex (H¯-TNT), dihydride-Meisenheimer complex (2H¯-TNT), 2-hydroxylamino-6-nitrotoluene (2HA6NT), 2,4-dinitrotoluene (24DNT), 2-nitrotoluene (2NT), toluene (Tol), tricarboxylic acid cycle (TCA cycle), nitrite (NO_2_ˉ); (**b**) β-ketoadipate pathway composed of *ortho*-catechol cleavage (above) and protocatechuate (below) branches. The degradation pathways of some xenobiotics (e.g., nitroaromatics, benzene, etc.) converge in catechol, while others (e.g., phthalate, *p*-cresols) can converge in protocatechuate. TNT degradation pathways could also converge in the latter (TNT?). Unique enzymes of TNT isolates are shown in red letters. Enzymes involved in *ortho*-catechol branch are catechol 1,2-dioxygenase (C1,2O), *cis*,*cis*-muconate lactonizing enzyme (MLE), and muconolactone isomerase (MI). Enzymes involved in protocatechuate branch are protocatechuate 3,4-dioxygenase (P3,4O), β-carboxy-*cis*,*cis*-muconate lactonizing (CMLE), and γ-carboxy-muconolactone decarboxylase (CMD). Enzymes shared by both branches are β-ketoadipate enol-lactone hydrolase (ELH), β-ketoadipate succinyl-CoA transferase (TR), and β-ketoadipyl-CoA thiolase (TH). β-subunit (β).

In order to make better sense of the results obtained (specifically for the xenobiotic TNT), we compiled experimental and theoretical information retrieved from the literature to reconstruct the main metabolic pathways involved in TNT degradation that would be part of the metabolic machinery of TNT-transforming pseudomonads. For this purpose, the TNT-degrading enzymes characterized elsewhere (see Section 2.8), along with those identified in this study, were mapped to the routes in which they are likely to participate. In the scheme (Figure 6a), TNT transformation occurs under aerobic conditions and begins with this compound entering the bacterial cell by passive diffusion, and then being reduced via nitroreduction (red arrows) or denitration (blue arrows) pathways [100]. In the former, nitroreductases and OYEs (and possibly also azoreductases) catalyze the reduction of one nitro group to hydroxylaminodinitrotoluenes (HADNTs), forming nitrosodinitrotoluenes (NoDNTs) as intermediates. Then, HADNTs are reduced by nitroreductases to aminodinitrotoluenes (ADNTs) [13,114], which can accumulate inside the cell or be removed from it through the MexEF-OprN efflux pumps [30,100]. Since it has been proposed that these pumps could directly extrude TNT, we hypothesize that they could also extrude ADNTs. Furthermore, regarding nitroreduction, it has been observed that some TNT derivatives can react among themselves, forming recalcitrant azoxynitrotoluenes, which can be even more toxic than TNT itself [13].

TNT can also be reduced via the denitration pathway (blue arrows), where XenB is the only participating enzyme that has been reported so far in the genus *Pseudomonas*. XenB reduces the TNT aromatic ring to the hydride-Meisenheimer complex (Hˉ-TNT), which is then reduced to the dihydride-Meisenheimer complex (2Hˉ-TNT) by the same enzyme. This latter complex undergoes abiotic rearomatization, with the subsequent production of 2-hydroxylamino-6-nitrotoluene (2HA6NT) and nitrite release [100]. Alternatively, Hˉ-TNT can also be transformed into 2,4-dinitrotoluene (24DNT), which could subsequently be converted into toluene (Tol) and enter into the TCA cycle through a series of degradation steps. Since it has been recently reported that the protocatechuate 3,4-dioxygenase (P3,4O) enzyme of the β-ketoadipate pathway increases the degradation of TNT and 4ADNT in *Buttiauxella* sp. S19-1 [11], we propose that the P3,4O enzyme and other enzymes of the β-ketoadipate pathway (encoded by unique genes as mentioned earlier) could also participate in TNT transformation on TNT isolates. In Figure 6a, green arrows indicate that TNT-degrading enzymes (e.g., nitroreductases, OYEs, etc.) produced unidentified TNT metabolites that could enter into the β-ketoadipate metabolic pathway, to be later degraded via the catechol *ortho*-cleavage and/or the protocatechuate branches (Figure 6b), ultimately generating succinyl-CoA and acetyl-CoA, both metabolites of the TCA cycle.

## 4. Conclusions

The numerous applications of 2,4,6-trinitrotoluene (TNT) for both civil and military uses have steadily increased its presence as an environmental pollutant, leading to an urgent need for the development of remediation alternatives. Consequently, there is an ongoing search for novel microorganisms with enhanced metabolic capabilities for degrading this and other xenobiotics that could be used in bioremediation strategies. In this study, the comparative genomic approach used revealed the potential for bioremediation encoded in three novel TNT-transforming *Pseudomonas* isolated from Deception Island, an active volcano from Antarctica, which represents one of the most unique ecosystems of the world and a source of unknown microbiological diversity. Interestingly, despite the presence of fewer complete metabolic modules than in the *Pseudomonas* species to which they were compared, the number of annotated enzymes involved in xenobiotic degradation in their genomes is similar to that of species with high degradative versatility (e.g., *P. veronii* Pvy). Among the species compared in this study, the abundances of catabolic enzymes for degradation of TNT, fluorobenzoate, and bisphenol A are the highest in TNT11, TNT3, and TNT19, respectively. While we have previously experimentally demonstrated the transformation of the former compound by these isolates, the latter two have not yet been assessed and we consider them good candidates for future experimental studies on the degradation of halogenated and phenolic xenobiotics, whose biodegradation by Antarctic strains has not been previously reported.

More notably, we identified unique genomic features in our isolates, particularly genes encoding putative enzymes of central and peripheral metabolic pathways that are likely to be involved in xenobiotic degradation that are not found in other well-characterized TNT-transforming *Pseudomonas* species (e.g., *P. putida* strains). One of those genes (found in TNT3 and TNT19) encodes the β-subunit of protocatechuate 3,4-dioxygenase, a key enzyme in the β-ketoadipate metabolic pathway through which bacteria degrade aromatic compounds, whose involvement in TNT degradation has recently been demonstrated. Additionally, two unique genes that encode xenobiotic reductases (XenB and XenE) were also found in the same isolates. Of course, further research (e.g., gene knockout, transcriptomics, in vitro assays using recombinant enzymes) is required in order to elucidate the real role that this distinctive set of enzymes plays in our isolates regarding TNT degradation. Another interesting outcome of this study was the identification of what appears to be a gene cluster for TNT degradation in the genomes of *P. putida* strains JLR11 and KT2440, which to our knowledge, has not been previously described as such. Finally, since our results also indicate that TNT isolates correspond to novel species of non-pathogenic *Pseudomonas*, besides contributing to a better understanding of the diversity and xenobiotics metabolism of Antarctic bacteria, we think that it is worthwhile to further study them and to assess their usefulness as bioremediation agents for in situ treatment of contaminated environments.

## Figures and Tables

**Table 1:** General features of TNT isolates’ draft genome assemblies. Gene counts correspond to PATRIC/RASTtk annotation results. Completeness metrics are the result of BUSCO and CheckM assessments.

Genome Feature	Isolate		
	TNT3	TNT11	TNT19
Contig count	138	718	92
L50 value	22	126	11
Contigs N50 (bp)	93,061	13,749	166,334
Completeness (%)	99.2	96.8	99.2
Genome size (bp)	6,458,871	5,861,354	6,454,788
GC content (%)	58.55	60.44	58.60
Total genes	6185	6345	6385
Total number CDSs	6120	6280	6322
rRNA genes	5	6	5
tRNA genes	60	59	58
Proteins with functional assignment	4751	4943	4644
Hypothetical proteins	1369	1337	1678

**Table 2:** Putative enzymes potentially involved in 2,4,6-trinitrotoluene (TNT) transformation that are present in TNT isolates.

				Isolate		
		Route	Enzyme	TNT3	TNT11	TNT19
Central metabolic pathway	β-ketoadipate pathway	Protocatechuate	P3,4O (β-chain)	*Yes*	Yes (2 isoenzymes)	*Yes*
			CMD	*Yes* *(isoenzyme 1)*	*Yes* *(isoenzyme 1)*	*Yes* *(isoenzyme 1)*
				*Yes* *(isoenzyme 2)*	Yes (isoenzyme 2)	Yes (isoenzyme 2)
		*Ortho*-catechol	C1,2O	*Yes*	Yes	*Yes*
			MLE	*Yes*	Yes	*Yes*
		*Ortho*-catechol and protocatechuate	TR	*Yes* *(A chain)*	Yes (A chain)	*Yes* *(A chain)*
				*Yes* *(B chain)*	*Yes* *(B chain)*	*Yes* *(B chain)*
			TH	*Yes*	Yes	*Yes*
Peripheral metabolic pathway	TNT degradation pathway	Nitroreduction	NitroR4	Yes	Yes	Yes
			NitroR5	Yes	Yes	Yes
			NitroR6	-	Yes	-
			AzoR-a	Yes	Yes	Yes
			AzoR-b	Yes	Yes	-
			AzoR-c	Yes	-	-
		Nitroreduction and denitration	XenA	-	Yes	-
			XenB	*Yes*	Yes	*Yes*
			XenC	-	Yes	-
			XenE	*Yes*	Yes	*Yes*

This table shows the presence (yes) and absence (-) of central and peripheral enzymes found in TNT isolates. “*Yes*” in italics indicates enzymes encoded by unique genes. Protocatechuate 3,4-dioxygenase (P3,4O), γ-carboxy-muconolactone decarboxylase (CMD), catechol 1,2-dioxygenase (C1,2O), *cis*,*cis*-muconate lactonizing enzyme (MLE), β-ketoadipate succinyl-CoA transferase (TR), β-ketoadipyl-CoA thiolase (TH), nitroreductase (NitroR), azoreductase (AzoR), xenobiotic reductase A (XenA), xenobiotic reductase B (XenB), xenobiotic reductase C (XenC), xenobiotic reductase E (XenE).

## Data Availability

The data presented in this study are available in this article and Supplementary Materials. The draft genome sequences of *Pseudomonas* sp. TNT3, TNT11, and TNT19 have been deposited in GenBank database (NCBI) under the accession number WFGV00000000, JAKNRV000000000, and JAKNRW000000000, respectively.

## Supplementary Materials

The following supporting information can be downloaded at: https://www.mdpi.com/article/10.3390/genes13081354/s1, Figure S1: Circular genome maps of TNT isolates; Figure S2: Draft genomes completeness of TNT isolates; Figure S3: Counts of COG categories in TNT isolates; Figure S4: KEGG modules completeness per genome as predicted by MicrobeAnnotator; Figure S5: Metabolic potential comparison among the pseudomonads used in this study; Figure S6: Pangenome of the 24 *Pseudomonas* spp. analyzed; Figure S7: Metabolic functions encoded by unique genes in TNT isolates; Figure S8: Average nucleotide identity heat map based on MUMmer algorithm (ANIm) among the 24 pseudomonads considered in this study; Figure S9: Genomic islands (GIs) predicted in the genomes of TNT isolates; Figure S10: Putative gene cluster for production of xanthoferrin in TNT19 isolate; Figure S11: Multiple sequence alignment of putative nitroreductases in some pseudomonads; Figure S12: Multiple sequence alignment of putative xenobiotic reductases in some pseudomonads; Figure S13: Multiple sequence alignment of putative azoreductases in some pseudomonads; Figure S14: Neighbor-ioining (NJ) cladogram depicting phylogenetic relationships among TNT-degrading enzymes; Table S1: Characteristics of the pseudomonads used for comparative genomic analysis in this study; Table S2: Enzymes encoded by genes of the “trinitrotoluene degradation” pathway found by PATRIC/RASTtk in TNT isolates genomes; Table S3: The pangenome of the 24 pseudomonads used in this study and groups of sub-pangenomes constructed with Roary; Table S4: Prophage regions found in the genomes of TNT-transforming bacteria; Table S5: Putative TNT-degrading enzymes found in TNT isolates. References [115,116,117,118,119,120,121,122,123,124,125,126,127,128] are cited in the Supplementary Materials.
