# Supplementary material for: Comparative Genomic Analysis of Antarctic Pseudomonas Isolates with 2,4,6-Trinitrotoluene Transformation Capabilities Reveals Their Unique Features for Xenobiotics Degradation
Source: Genes (Basel). 2022 Jul 28;13(8):1354. doi: 10.3390/genes13081354 (PMC9407559; doi:10.3390/genes13081354)
Supplement: Supplementary file 1 [file genes-13-01354-s001.zip › supplementary_figures2507.pdf]

genes

MDPI

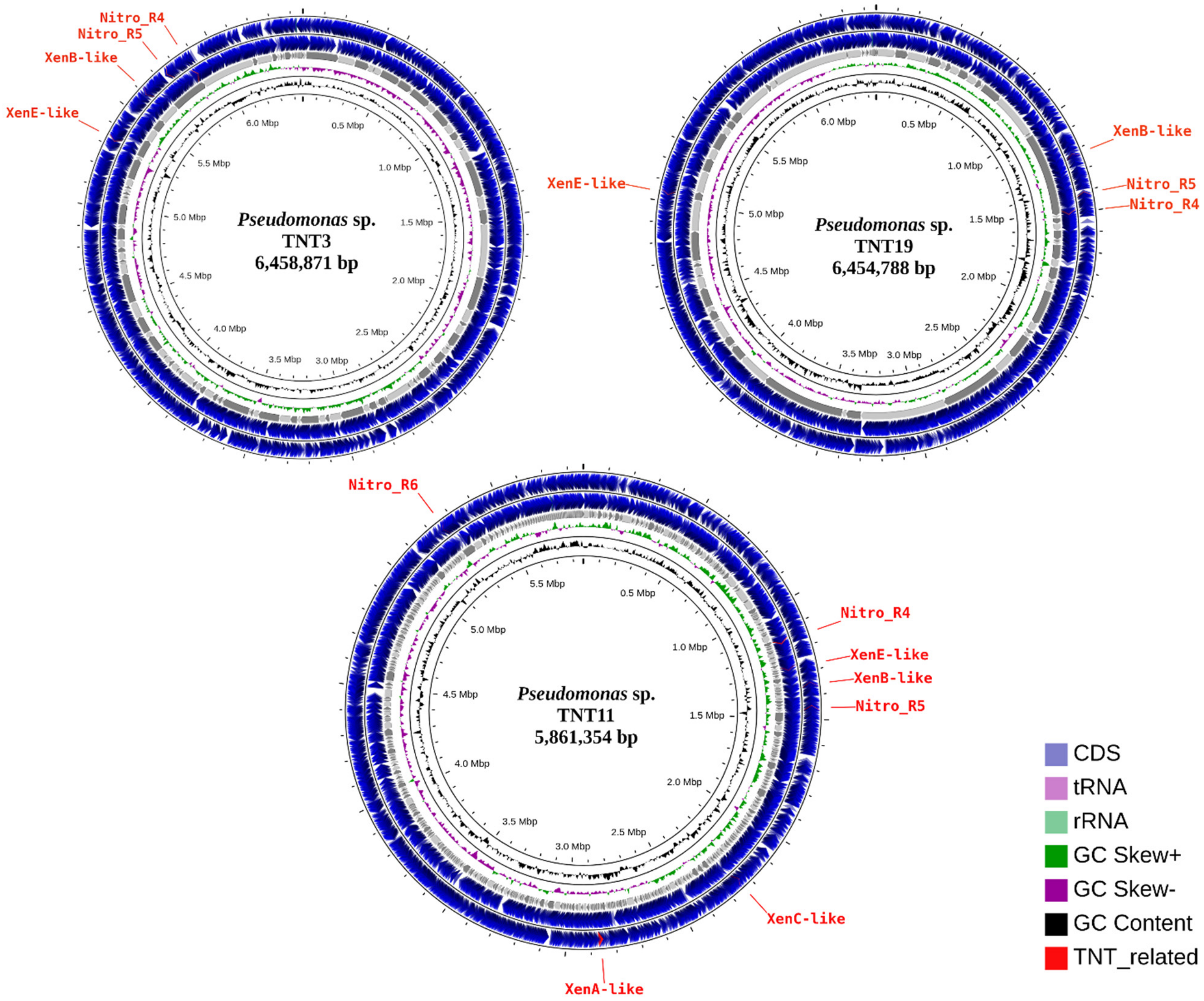

Figure S1. Circular genome maps of TNT isolates. Tracks from outermost to innermost showing protein coding sequences on reverse (1) and forward strands (2); contigs ordered by reference (3); average GC skew (4); GC content (5); genome scale in Mbp (6). The position in the genome for putative nitroreductases (Nitro_R4-R6) and xenobiotic reductases (XenA, XenB, XenC, XenE) are shown in red. The contigs of the three genomes were ordered using the genome of *P. fluorescens* Pf0-1 (the closest representative genome according to PATRIC's Similar Genome Finder) as reference. Representations were generated using CGView/Proksee (https://proksee.ca/).

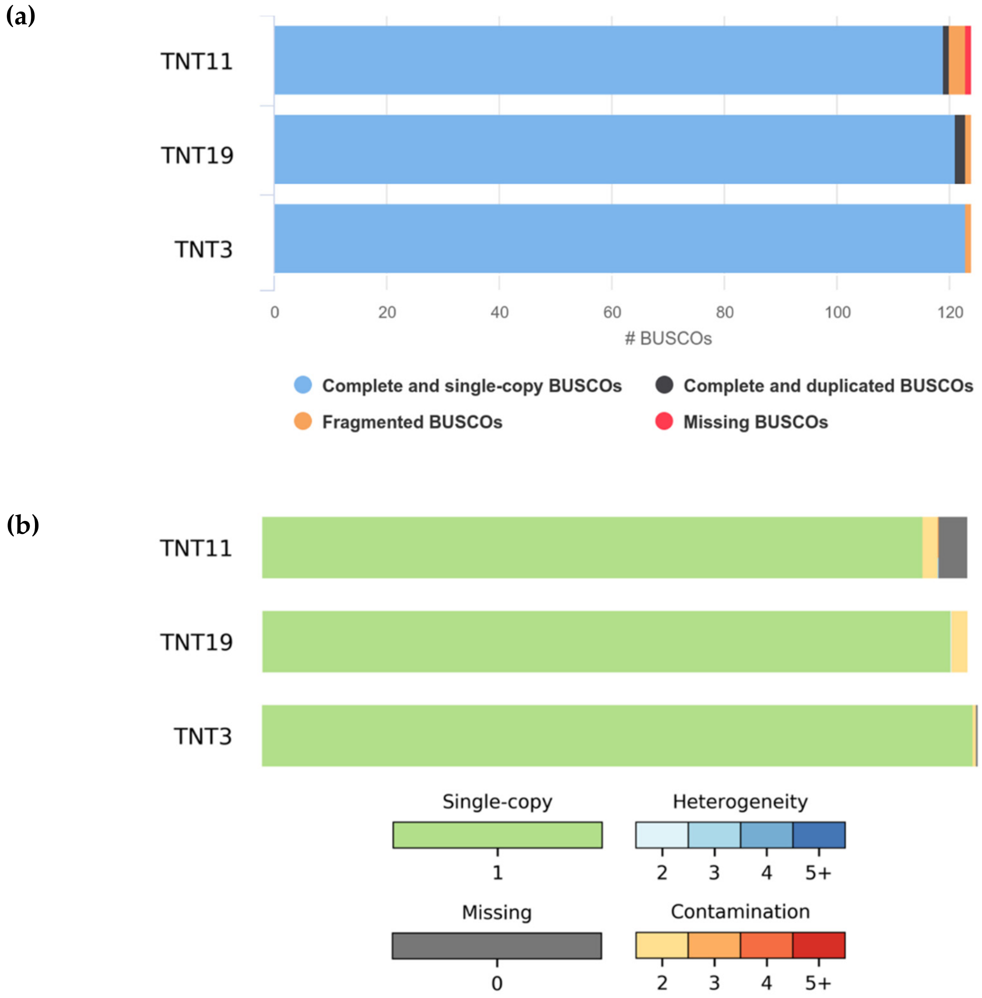

Figure S2. Draft genomes completeness of TNT isolates. **(a)** Number of complete, missing, and fragmented Universal Single-Copy Orthologs (BUSCOs) found in genome assemblies; **(b)** CheckM completeness and contamination assessment results of draft genome assemblies.

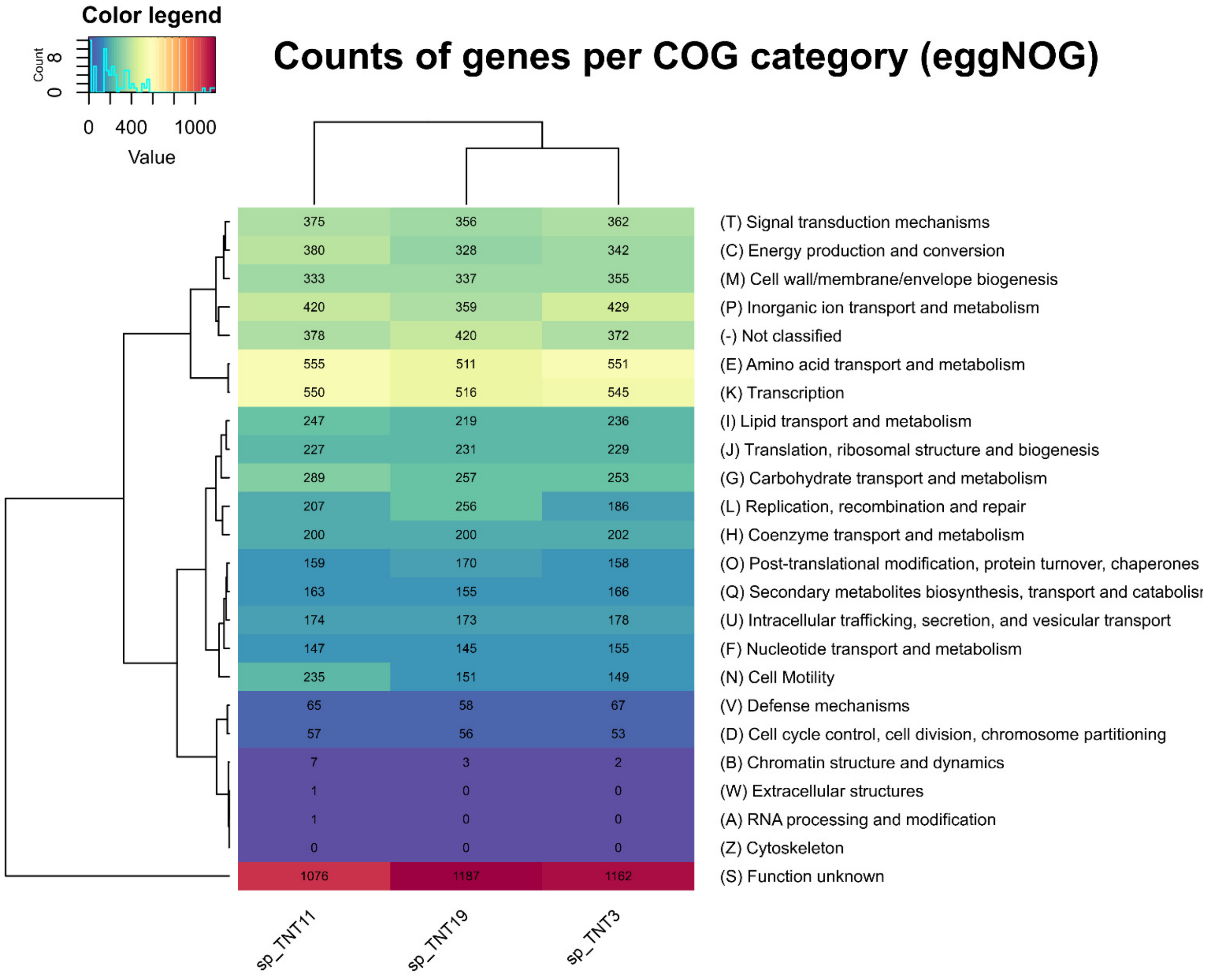

Figure S3. Counts of COG categories in TNT isolates. Total count of genes associated with COG functional categories per genome, as assigned by eggNOG-mapper.

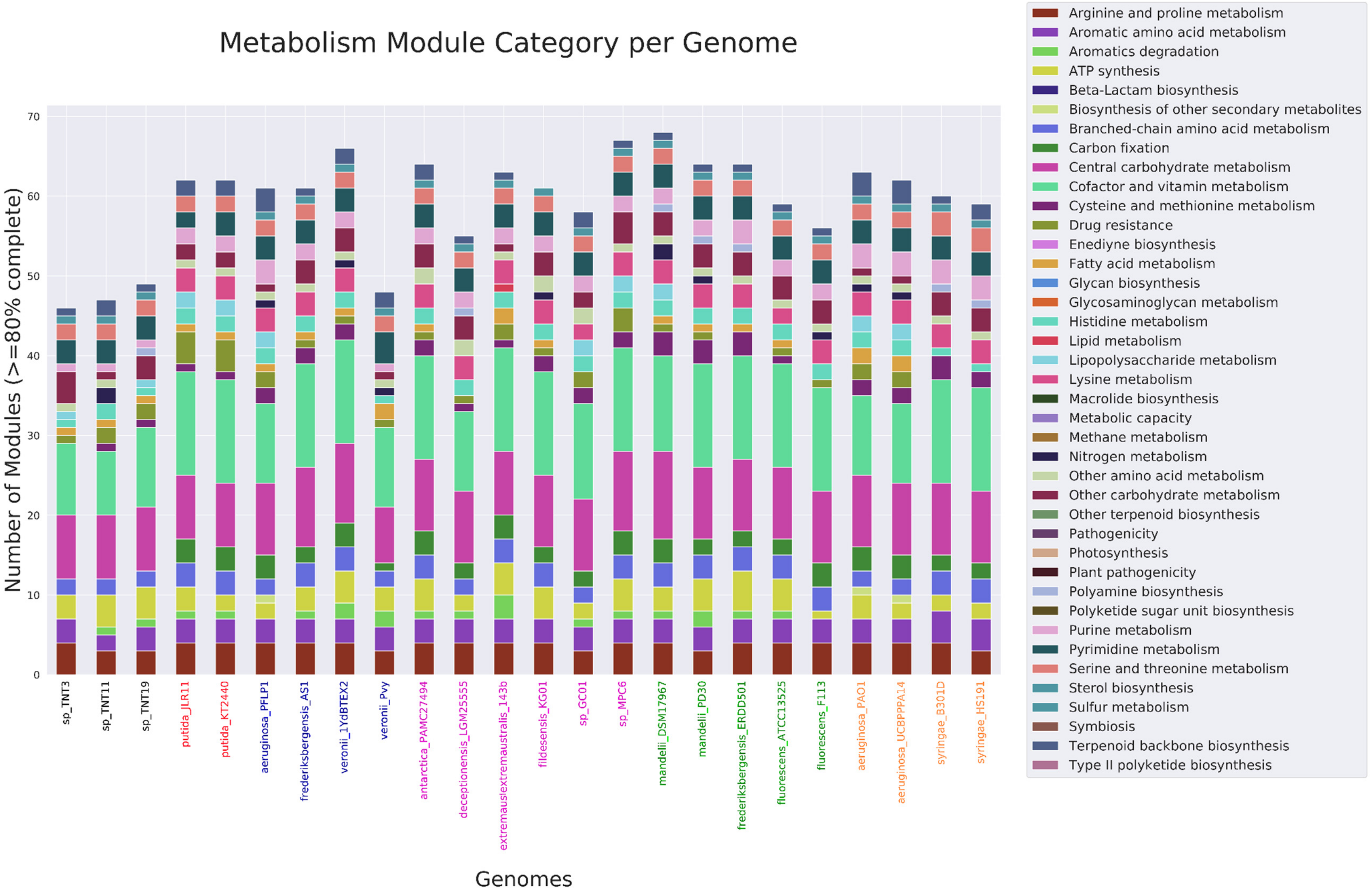

Figure S4. KEGG modules completeness per genome as predicted by MicrobeAnnotator. The bar plot shows the number of metabolic modules (≥80% completeness) in the genomes included in the analysis. The different groups of pseudomonads are indicated in black (TNT isolates), red (Group 1), blue (Group 2), magenta (Group 3), green (Group 4), and orange (Group 5).

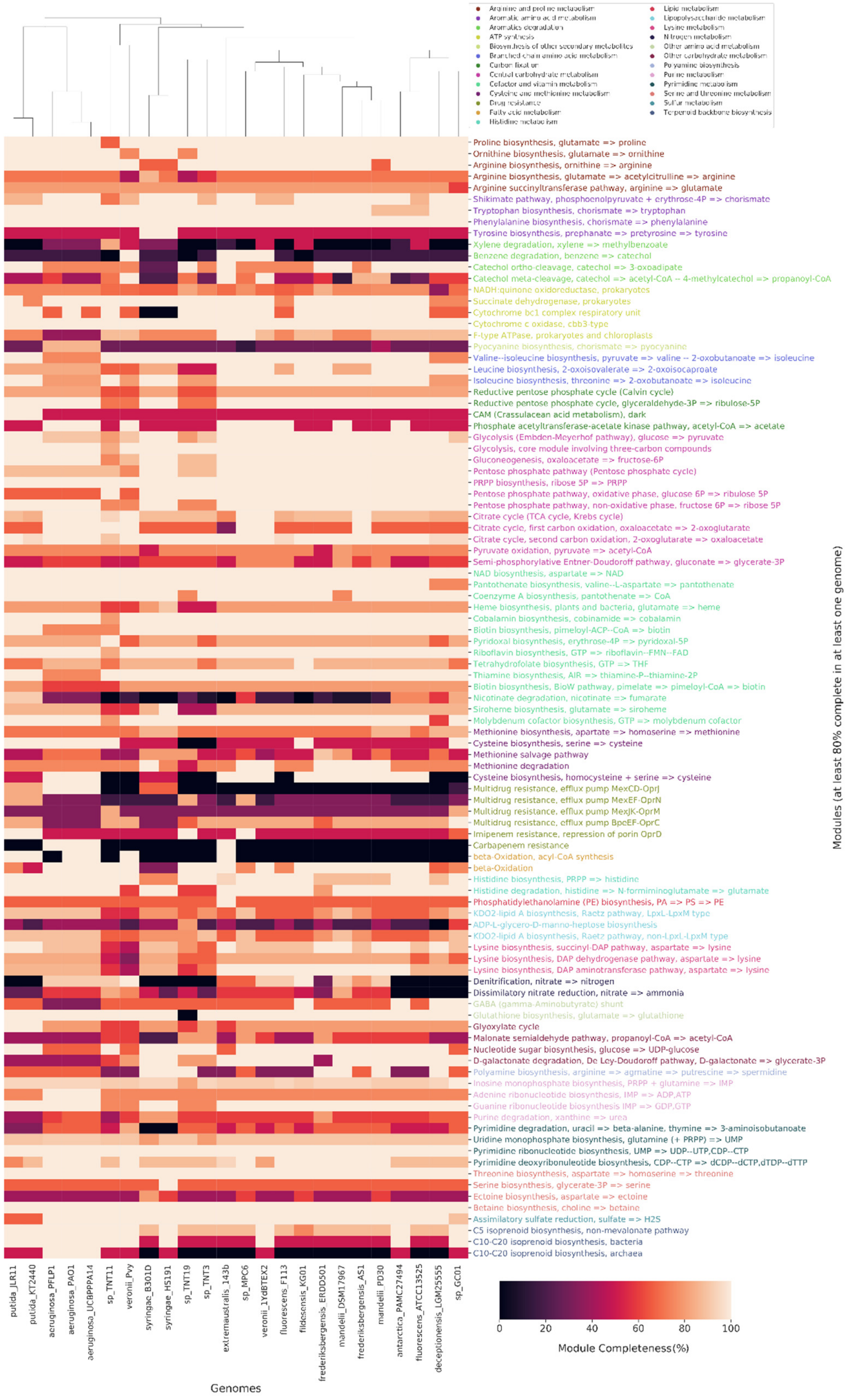

Figure S5. Metabolic potential comparison among the pseudomonads used in this study. The heat map shows the completeness of KEGG metabolic modules per genome.

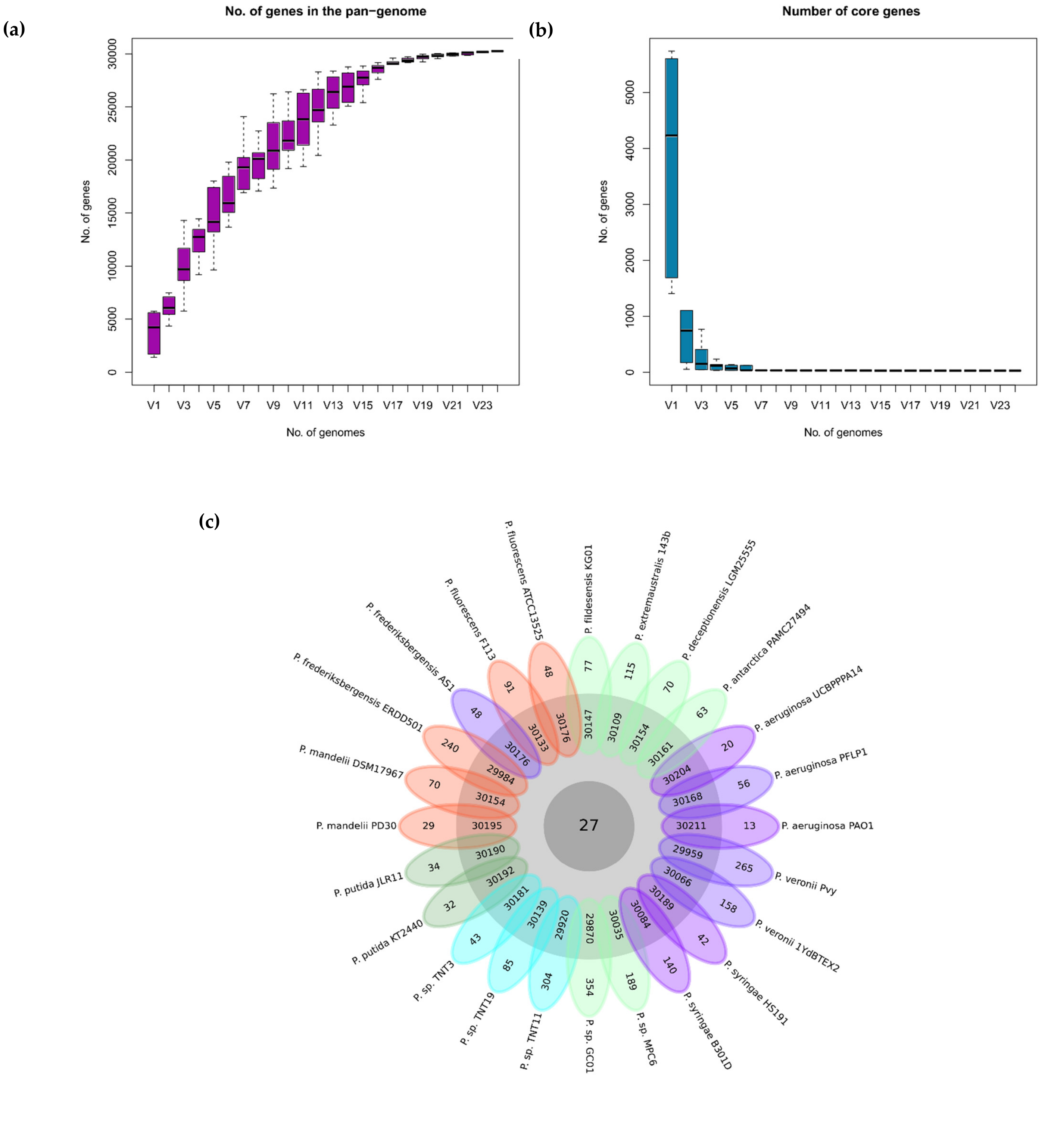

Figure S6. Pangenome of the 24 *Pseudomonas* spp. analyzed. **(a)** Cumulative number of genes in pangenome and **(b)** core genome as a function of the genomes included in the analysis; **(c)** Flower plot diagram showing core, accessory, and unique genes across all species. The inner circle shows the number of genes common to all species (hard core genes), petals show accessory genes (inside the circle) and unique genes (outside the circle)

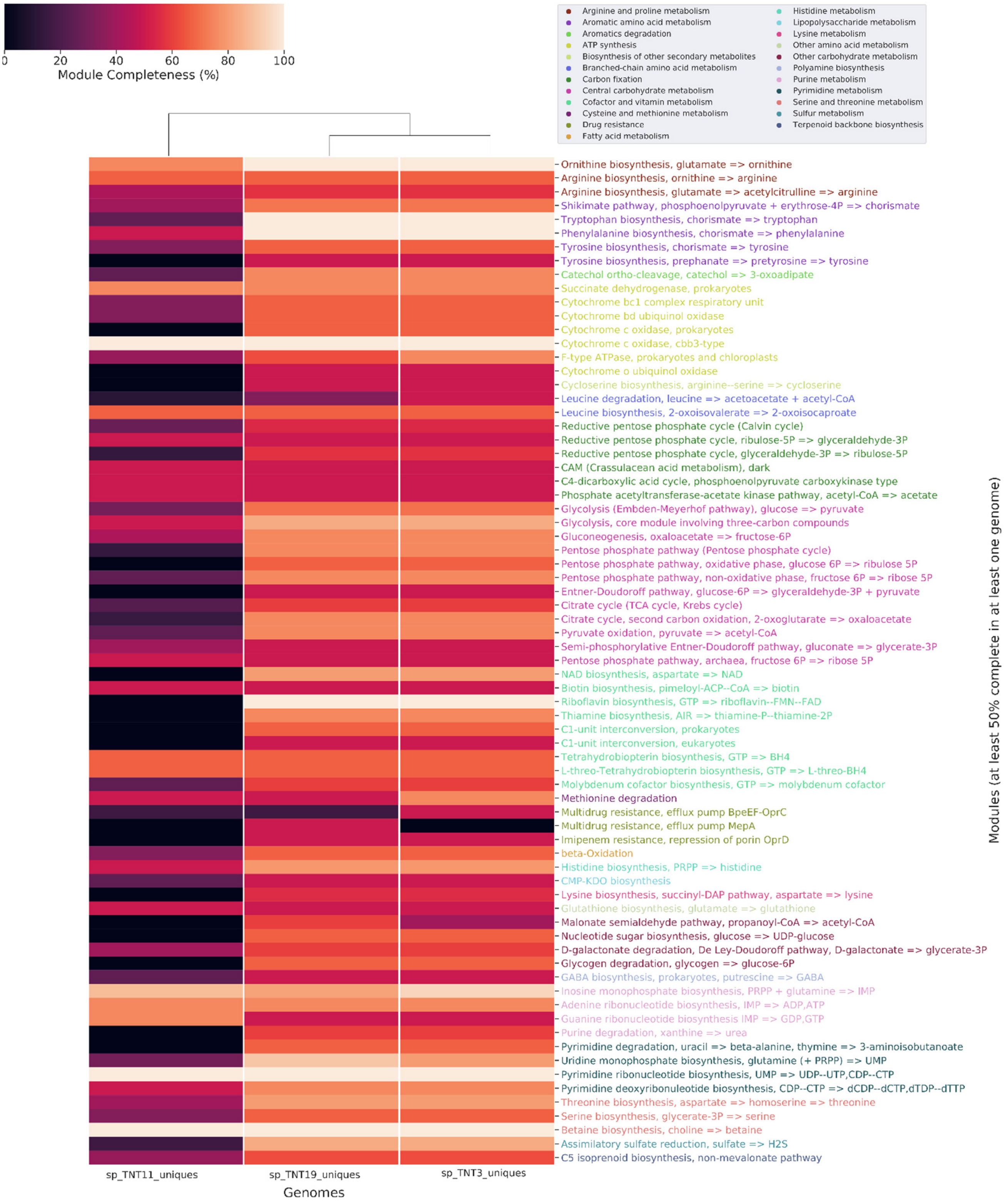

Figure S7. Metabolic functions encoded by unique genes in TNT isolates. The genes in these modules were considered as unique by Roary when querying the sub-pangenome of Group 1 (TNT isolates plus *P. putida* strains KT2440 and JLR11).

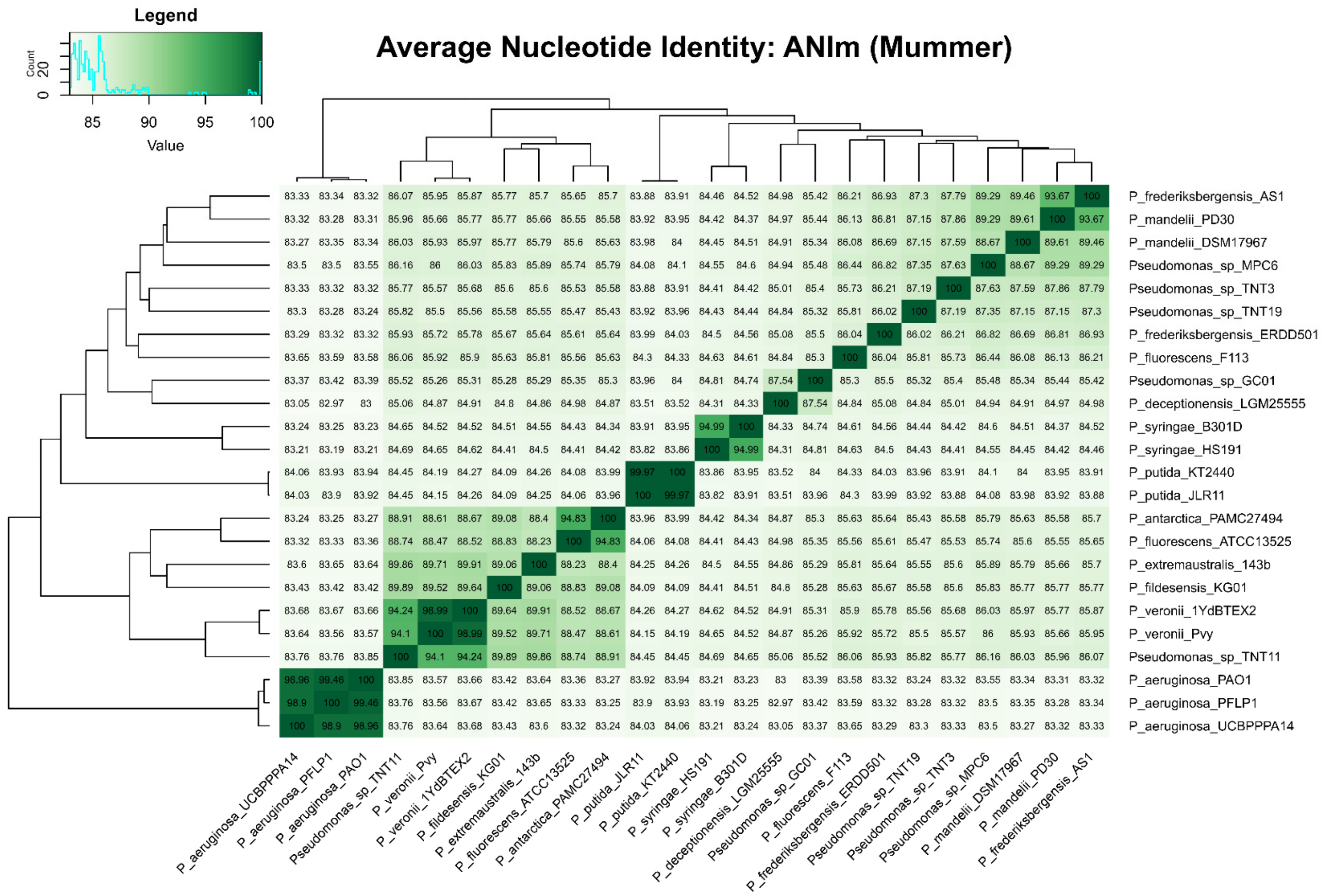

Figure S8. Average nucleotide identity heat map based on MUMmer algorithm (ANIm) among the 24 pseudomonads considered in this study. ANI values were calculated with PyANI after blastn alignment. Values are shown as percentages (%) of aligned nucleotides. ANI values above 95% between two genomes indicate they belong to the same species.

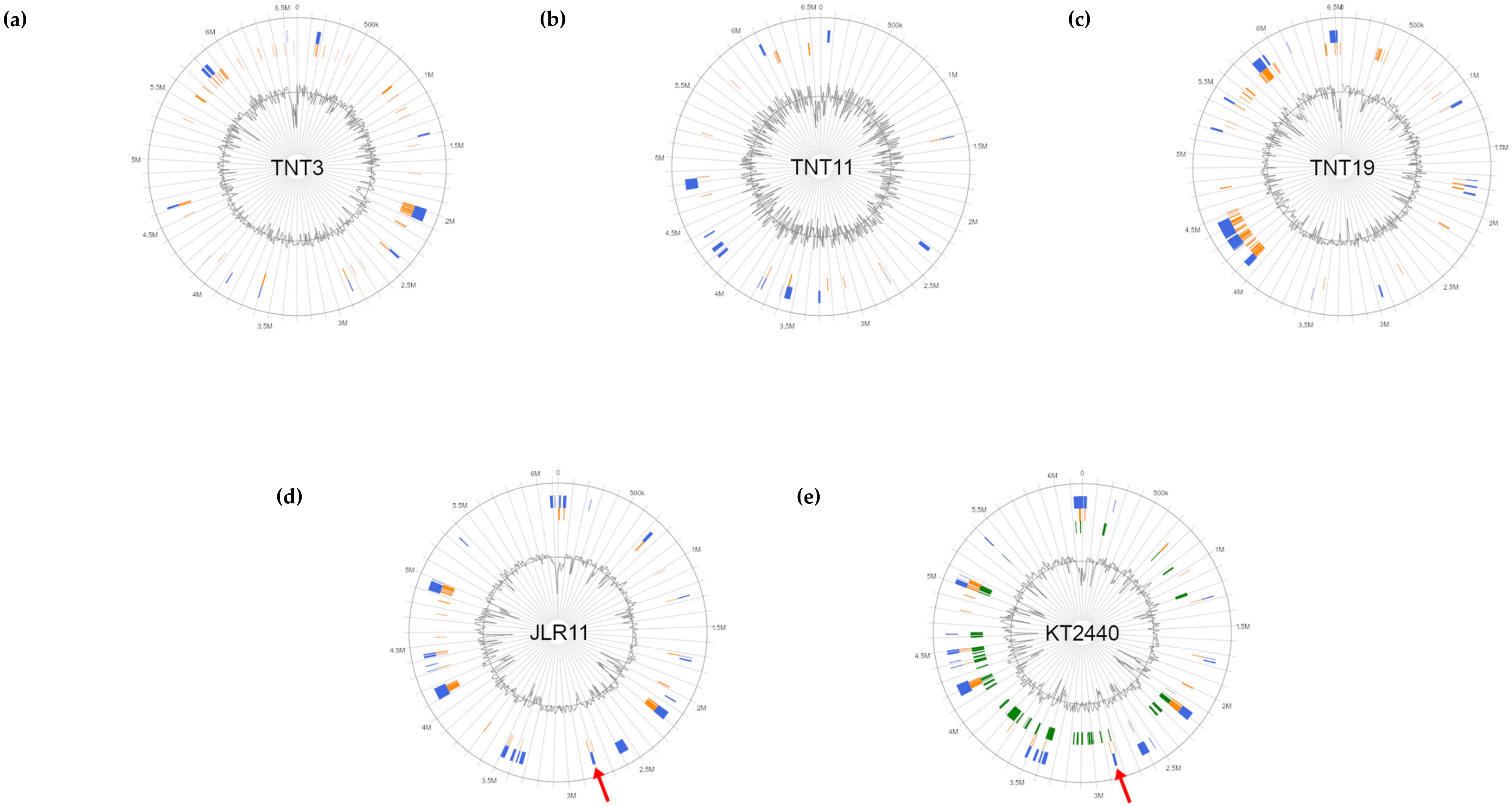

Figure S9. Genomic islands (GIs) predicted in the genomes of TNT isolates. **(a)** TNT3; **(b)** TNT11; **(c)** TNT19; **(d)** JLR11, and **(e)** KT2440. GIs were predicted by IslandPath-DIMOB (blue), SIGI-HMM (yellow), and IslandPick (green) methods. Red arrows indicate the putative gene cluster of TNT-degrading enzymes in strains JLR11 and KT2440.

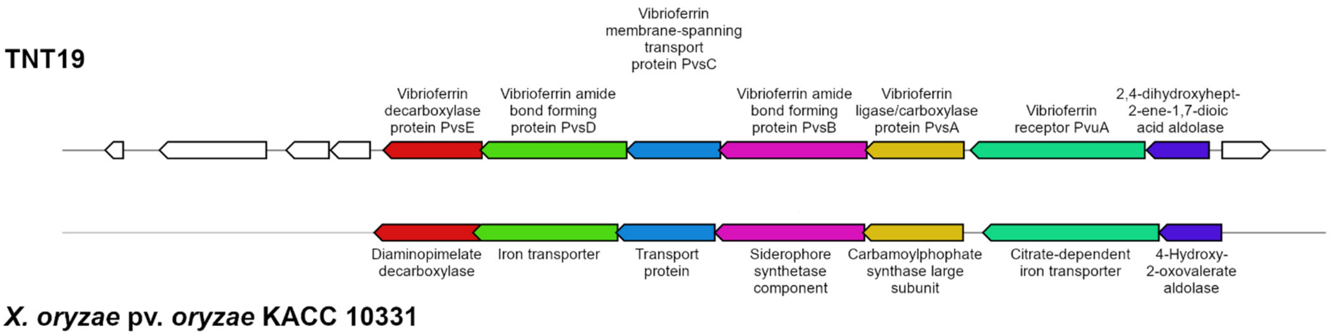

Figure S10. Putative gene cluster for production of xanthoferrin in TNT19 isolate. This putative gene cluster in TNT19 (above) was predicted to share 100% similarity to that of *X. oryzae* pv. *oryzae* KACC 10331 (below).

(a)

```
NitroR2_JLR11          MEALDALLNRVSVPRLTDPAPNAAQREALFQAALRAPDHGQLRPWRFLTIEGQGREKLGELFAEAVQH-KGDASQAALDKARAMPLRAPLLIVVVARLQDHFKVPKSEQR  110
NitroR2_KT2440         MEALDALLNRVSVPRLTDPAPNAAQREALFQAALRAPDHGQLRPWRFLTIEGQGREKLGELFAEAVQH-KGDASQAALDKARAMPLRAPLLIVVVARLQDHFKVPKSEQR
NitroR4_TNT11          MQALDALLNRVSVPRLLEPAPTQEQRDVLFAAAMRAPDHGQLRPWRFFTVEGAAREQLGTLLVEAARLQDPDAPQAALDKAQNGPMRAPLVVVVIARLQEHFKVPKSEQL
NitroR4_TNT19          MQALDVLLNRVSVPRLLEPAPTAEQREVLFCAAMRAPDHGHLQPWRFLTVEGAAREQMGELLAEAAKLQDSEVTEAAVDKARNGPLRAPLVVVVIARLQDHVKYPKSEQM
NitroR4_TNT3           MQALDALLNRVSVPRLLEPAPTAEQREVLFAAAMRAPDHGHLQPWRFLTVEGAAREQMGELLAEAAKLQDSEVSDAALDKARNGPLRAPLVVVVIARLQDHVKYPKSEQL
P_fluorescens_ABAC62   MQALDALLNRVSVPRLLEPAPTQEQRDVLFAAAMRAPDHGQLRPWRFLTVEGAAREQMGTLLAEAARLQDADAPQAAIDKAQNGPLRAPLVVVVIARLQEHFKVPKSEQL
Pseudomonas_sp_MF4836  MEALDVLLNRVSVPRLLDPAPDAAQREALFAAALRAPDHGQLRPWRFLTVEGAAREQMGELLAEAVQLQGGEVTQAALDKARAMPLRAPLVVVVVARLQEHFKVPKSEQL
                       *:***.********** :***   **:.** **:******:*:****:*:** .**::* *:.**.:    :..:**:***:  *:****::**:****:*.* *****

NitroR2_JLR11          LAAGCAAHGILIAAHAQGIGAVWRTGDMAFDAHVHKGLGLAENEELIGYLYVGTPLTEPRTAPVLETADFVSSWGE--  187
NitroR2_KT2440         LAAGCAAHGILIAAHAQGIGAVWRTGDMAFDAHVHKGLGLAENEELIGYLYVGTPLTEPRTAPVLETADFVSSWGE--
NitroR4_TNT11          LAAACAAHGILLAAYAQGIGAVWRTGDLSYSAHVAKGLGLTAGEEVIGFLYLGTPQNPPRTAPKEDLAAFVQAWPG-L
NitroR4_TNT19          LAAGCAAHGILLAAYAQGIGAVWRTGDLAYSPHLAKGLGLAEGEEVIAFLYLGTPQKEPRVAEKVDLAEFVSAWPGKS
NitroR4_TNT3           LAAGCAAHGILLAAYAQGIGAVWRTGELAYSAHVAKGLGLAEGEEVIAFLYLGTPQKEPRTAEKVDLAEFVSAWPR-H
P_fluorescens_ABAC62   LAAACAAHGILLAAYAQGIGAVWRTGELSYSAHVAKGFGLAADEEVIGFLYLGTPQNPPRVAPKEDLAAFVQAWPG-R
Pseudomonas_sp_MF4836  LAAGCAAHGILLAAYAQGIGAVWRTGELSYSAHVAKGLGLGEGEEIIAFLYLGTPQNEPRVAPKVDTAEFVSAWSGQG
                       ***.*******:**:***********::::..*: **:**  .**:*.:**:*** . **.*   : * **.:*
```

(b)

```
NitroR5_TNT11          MSTNPRAADYPINPQFTDRWSPRAFTGASIPQETLLSFFEAARWAPSAYNSQPWRFLYARRDTPDWERFLGLLNEFNRNWAQHASALVIIASKTDFIAPGASEETPALWH  110
NitroR5_TNT19          MSANPRVADYAIHPQFTDRWSPRAFTGEAIPEETLLSFFEAARWAPSAYNSQPWRFLYARRDTPNWERYLGLLNEFNRSWAQHASALIIVISKTSFTAPGASEETPALWH
NitroR5_TNT3           MSNNPRVADYVIHPQFTDRWSPRAFTCEAIPEETLLSFFEAARWAPSAYNSQPWRFLYARRDTPNWERYLGLLNEFNRGWAQHASALVIVISKTTFTAPGATEETPALWH
P_syringae_pv_tomato   MSSNPRIADHPIDPQFTERWSPRAFSGESIEQETLLSFFEAARWAPSAYNSQPWRFLYARRDTPNWERYLGLLNEFNRNWAQHAALVIVISKTTFTAPGATEEGPALWH
Pseudom_FW507-12TSA    MSANPRVADHAIHPQFTERWSPRAFTAETIPEKTLLSFFEAARWAPSAYNSQPWRFLYARRDTPNWERFLGLLNEFNRGWAQHASALVIVISKTTFAAPGASEETPALWH
                       ** *** **: *.****:*******:  :* ::*******************************:***:*********.*****:**:*: *** * ****:** *****

NitroR5_TNT11          TFDTGSAWGYLALQASLSGWHTHGMAGFDQELTRKELKVPQGYALHAAVAIGKLGDKSTLPEYLQGRETPSPRKPLSELVSQGDFSL  197
NitroR5_TNT19          TFDTGSAWGHLALQASISGWHTHGMAGFDQALTRKELNIPEGYALHAAVAVGKLGDKATLAQYLQSRELPSPRRPLAELAAEGDFTL
NitroR5_TNT3           TFDTGSAWGHLALQASISGWHTHGMAGFDQELTRKELNIPEGYSLHAAVAVGKLGDKSTLADYLQAREVPSPRRPLSELVAEGDFSL
P_syringae_pv_tomato   TFDTGSAWGYLALQASLSGWHTHGMAGFDQELTRKELNIPEGYALHAAVAIGKLGDKSTLPEYLQGREVPSPRKPLDELAAEGDFSL
Pseudom_FW507-12TSA    TFDTGSAWGHLALQASLSGWHTHGMAGFDQELTRKELKIPDGYALHAAVAVGKLGDKSTLAEYLQAREVPSPRRPLSETVAEGDFNL
                       *********:******:************* ******::*:**:******:******:**.:***.** ****:** *  .::***.*
```

(c)

```
NitroR6_TNT11          MSGIAEVIKSRISANSYDTARQLTDAQIAELIDLATHAPSAFNLQNWRFVAVRSTEGKARLLPLAFGQQKVVDAAVTFIVCGTLNPQATLPASLKPTLDAGIIDQAIYDM  110
P_mucidolens           MSAIAEVIKSRVSANSYDTERSLTIAQITALVDLATHAPSAFNLQNWKFLAVDSIQAKARLLPLAFGQQKVVDAAVTFIVCGTLNPHLTLPDALKPTLDAGIIDQSVYDM
                       **.********:******* *.** ***: *:***************:*:** * :.*****************************: *** :***********::***

NitroR6_TNT11          WLGAAQGMYAQNPQLQRDEAIRSGSLAAMTLMLAAKGQGLVSTPMIGFDQSAVAQAFGLSDTEIPVMLVTVGYPGATNWPQKPRKAVNDVLQFV  204
P_mucidolens           WVGAAQGMYKENAQLQRDEAIRSGSLAAMTLMLAAKGQGLVSTPMIGFDQVAVAKEFGLSDEEVPVMLITVGYPGVANWPQKPRKEVSNVLQFV
                       *:******* :*.******************************** ***: ***** *:****:******.:******** *.:*****
```

Figure S11. Multiple sequence alignment of putative nitroreductases in some pseudomonads. **(a)** NitroR2 from *P. putida* strains KT2440 and JLR11, NitroR4 from TNT isolates, Putative NAD(P)H nitroreductase from *P. fluorescens* ABAC62 (UniProtKB: A0A656YN39), putative NAD(P)H nitroreductase from *Pseudomonas* sp. MF4836 (UniProtKB: A0A1T1HZT3); **(b)** NitroR5 from TNT isolates, Nitroreductase family protein from *P. syringae* pv. *tomato* ATCC BAA-871 (UniProtKB: Q87Y84) and *Pseudomonas* sp. FW507-12TSA (UniProtKB: A0A2K4IRJ3); **(c)** NitroR6 from TNT isolates and Nitroreductase from *P. mucidolens* (UniProtKB: A0A1H2MQ87). Residues involved in FMN binding are shown in red.

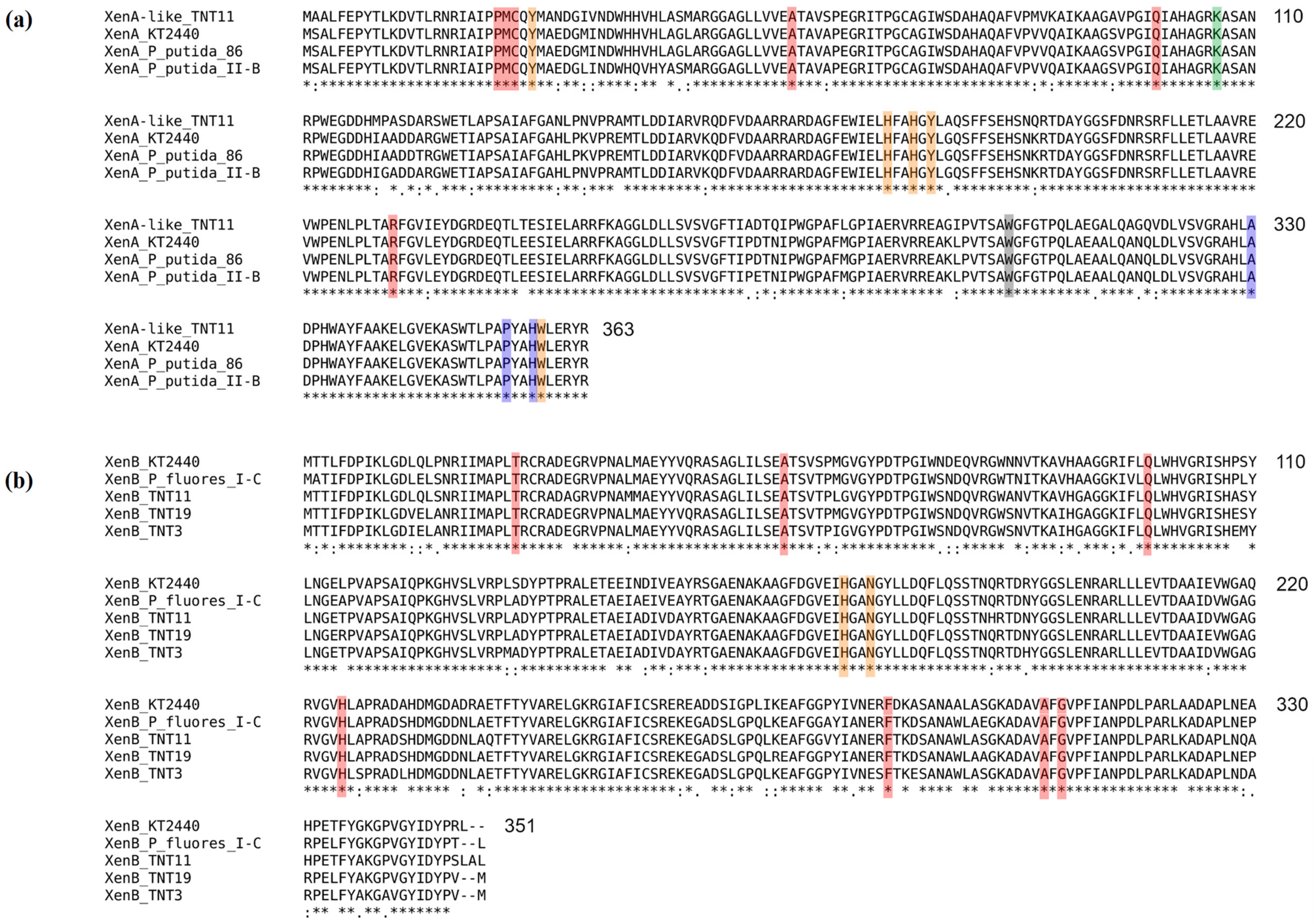
(a)
XenA-like_TNT11 MAALFEPYTLKDVTLRNRIAIPPMCQYMANDGIVNDWHHVHLASMARGGAGLLVVEATAVSPEGRITPGCAGIWSDAHAQAFVPMVKAIKAAGAVPGIQIAHAGRKASAN 110
XenA_KT2440 MSALFEPYTLKDVTLRNRIAIPPMCQYMAEDGMINDWHHVHLAGLARGGAGLLVVEATAVAPEGRITPGCAGIWSDAHAQAFVPVVQAIKAAGSVPGIQIAHAGRKASAN
XenA_P_putida_86 MSALFEPYTLKDVTLRNRIAIPPMCQYMAEDGMINDWHHVHLAGLARGGAGLLVVEATAVAPEGRITPGCAGIWSDAHAQAFVPVVQAIKAAGSVPGIQIAHAGRKASAN
XenA_P_putida_II-B MSALFEPYTLKDVTLRNRIAIPPMCQYMAEDGLINDWHQVHYASMARGGAGLLVVEATAVAPEGRITPGCAGIWSDAHAQAFVPVVQAIKAAGSVPGIQIAHAGRKASAN
XenA-like_TNT11 RPWEGDDHMPASDARSWETLAPSAIAFGANLPNVPRAMTLDDIARVRQDFVDAARRARDAGFEWIELHFAHGYLAQSFFSEHSNQRTDAYGGSFDNRSRFLLETLAAVRE 220
XenA_KT2440 RPWEGDDHIAADDARGWETIAPSAIAFGAHLPKVPREMTLDDIARVKQDFVDAARRARDAGFEWIELHFAHGYLGQSFFSEHSNKRTDAYGGSFDNRSRFLLETLAAVRE
XenA_P_putida_86 RPWEGDDHIAADDTRGWETIAPSAIAFGAHLPKVPREMTLDDIARVKQDFVDAARRARDAGFEWIELHFAHGYLGQSFFSEHSNKRTDAYGGSFDNRSRFLLETLAAVRE
XenA_P_putida_II-B RPWEGDDHIGADDARGWETIAPSAIAFGAHLPNVPRAMTLDDIARVKQDFVDAARRARDAGFEWIELHFAHGYLGQSFFSEHSNKRTDAYGGSFDNRSRFLLETLAAVRE
XenA-like_TNT11 VWPENLPLTARFGVIEYDGRDEQTLTESIELARRFKAGGLDLLSVSVGFTIADTQIPWGPAFLGPIAERVRREAGIPVTSAWGFGTPQLAEGALQAGQVDLVSVGRAHLA 330
XenA_KT2440 VWPENLPLTARFGVLEYDGRDEQTLEESIELARRFKAGGLDLLSVSVGFTIPDTNIPWGPAFMGPIAERVRREAKLPVTSAWGFGTPQLAEAALQANQLDLVSVGRAHLA
XenA_P_putida_86 VWPENLPLTARFGVLEYDGRDEQTLEESIELARRFKAGGLDLLSVSVGFTIPDTNIPWGPAFMGPIAERVRREAKLPVTSAWGFGTPQLAEAALQANQLDLVSVGRAHLA
XenA_P_putida_II-B VWPENLPLTARFGVLEYDGRDEQTLEESIELARRFKAGGLDLLSVSVGFTIPETNIPWGPAFMGPIAERVRREAKLPVTSAWGFGTPQLAEAALQANQLDLVSVGRAHLA
XenA-like_TNT11 DPHWAYFAAKELGVEKASWTLPAPYAHWLERYR 363
XenA_KT2440 DPHWAYFAAKELGVEKASWTLPAPYAHWLERYR
XenA_P_putida_86 DPHWAYFAAKELGVEKASWTLPAPYAHWLERYR
XenA_P_putida_II-B DPHWAYFAAKELGVEKASWTLPAPYAHWLERYR
(b)
XenB_KT2440 MTTLFDPIKLGDLQLPNRIIMAPLTRCRADEGRVPNALMAEYYVQRASAGLILSEATSVSPMGVGYPDTPGIWNDEQVRGWNNVTKAVHAAGGRIFLQLWHVGRISHPSY 110
XenB_P_fluores_I-C MATIFDPIKLGDLELSNRIIMAPLTRCRADEGRVPNALMAEYYVQRASAGLILSEATSVTPMGVGYPDTPGIWSNDQVRGWTNITKAVHAAGGKIVLQLWHVGRISHPLY
XenB_TNT11 MTTIFDPIKLGDLQLSNRIIMAPLTRCRADAGRVPNAMMAEYYVQRASAGLILSEATSVTPLGVGYPDTPGIWSNDQVRGWANVTKAVHGAGGKIFLQLWHVGRISHASY
XenB_TNT19 MTTIFDPIKLGDVELANRIIMAPLTRCRADEGRVPNALMAEYYVQRASAGLILSEATSVTPMGVGYPDTPGIWSNDQVRGWSNVTKAIHGAGGKIFLQLWHVGRISHESY
XenB_TNT3 MTTIFDPIKLGDIELANRIIMAPLTRCRADEGRVPNALMAEYYVQRASAGLILSEATSVTPIGVGYPDTPGIWSNDQVRGWSNVTKAIHGAGGKIFLQLWHVGRISHEMY
XenB_KT2440 LNGELPVAPSAIQPKGHVSLVRPLSDYPTPRALETEEINDIVEAYRSGAENAKAAGFDGVEIHGANGYLLDQFLQSSTNQRTDRYGGSLENRARLLLEVTDAAIEVWGAQ 220
XenB_P_fluores_I-C LNGEAPVAPSAIQPKGHVSLVRPLADYPTPRALETAEIAEIVEAYRTGAENAKAAGFDGVEIHGANGYLLDQFLQSSTNQRTDNYGGSLENRARLLLEVTDAAIDVWGAG
XenB_TNT11 LNGETPVAPSAIQPKGHVSLVRPLADYPTPRALETAEIADIVDAYRTGAENAKAAGFDGVEIHGANGYLLDQFLQSSTNHRTDNYGGSLENRARLLLEVTDAAIDVWGAG
XenB_TNT19 LNGERPVAPSAIQPKGHVSLVRPLADYPTPRALETAEIADIVDAYRTGAENAKAAGFDGVEIHGANGYLLDQFLQSSTNQRTDNYGGSLENRARLLLEVTDAAIEVWGAG
XenB_TNT3 LNGETPVAPSAIQPKGHVSLVRPMADYPTPRALETAEIADIVDAYRTGAENAKAAGFDGVEIHGANGYLLDQFLQSSTNQRTDHYGGSLENRARLLLEVTDAAIDVWGAG
XenB_KT2440 RVGVHLAPRADAHDMGDADRAETFTYVARELGKRGIAFICSREREADDSIGPLIKEAFGGPYIVNERFDKASANAALASGKADAVAFGVPFIANPDLPARLAADAPLNEA 330
XenB_P_fluores_I-C RVGVHLAPRADSHDMGDDNLAETFTYVARELGKRGIAFICSREKEGADSLGPQLKEAFGGAYIANERFTKDSANAWLAEGKADAVAFGVPFIANPDLPARLKADAPLNEP
XenB_TNT11 RVGVHLAPRADSHDMGDDNLAQTFTYVARELGKRGIAFICSREKEGADSLGPQLKEAFGGVYIANERFTKDSANAWLASGKADAVAFGVPFIANPDLPARLKADAPLNQA
XenB_TNT19 RVGVHLAPRADSHDMGDDNLAETFTYVARELGKRGIAFICSREKEGADSLGPQLREAFGGPYIANERFTKDSANAWLAAGKADAVAFGVPFIANPDLPARLKADAPLNEP
XenB_TNT3 RVGVHLSPRADLHDMGDDNLAETFTYVARELGKRGIAFICSREKEGADSLGPQLKEAFGGPYIVNESFTKESANAWLASGKADAVAFGVPFIANPDLPARLKADAPLNDA
XenB_KT2440 HPETFYGKGPVGYIDYPRL-- 351
XenB_P_fluores_I-C RPELFYGKGPVGYIDYPT--L
XenB_TNT11 HPETFYAKGPVGYIDYPSLAL
XenB_TNT19 RPELFYAKGPVGYIDYPV--M
XenB_TNT3 RPELFYAKGAVGYIDYPV--M

(c)

```
_JLR11          MTQSELFKTTQLGPYTLKNRIVLPPLTRSRSSQPGNIANDLMATYYRQRSGAGFMVTEGTQIEPRGQGYAWTPGIHSPEQIQGWRKVTDAVH
_KT2440         MTQSELFKTTQLGPYTLKNRIVLPPLTRSRSSQPGNIANDLMATYYRQRSGAGFMVTEGTQIEPRGQGYAWTPGIHSPEQIQGWRKVTDAVH
XenC_TNT11      MTHSVLSTPIQLGHHTLKNRVVLPPLTRQRSAQPGDIATELMAEYYRQRASAGFMVTEGTQIEPRGQGYAWTPGIYTPAQIDGWRTVTDAVH
                **:* *  ..  *** :*****:*******.**:***:**.:***  *****:.************************::* **:***.******

XenC_JLR11      TSLQPGGEAPVAPSAIRADNVKVFIETGPGTGALADPSTPRALSTKEVKELVKLYAQAARNALDAGFDGVELHCANGYLVNQFISAHTNQRD
XenC_KT2440     TSLQPGGEAPVAPSAIRADNVKVFIETGPGTGALADPSTPRALSTKEVKELVKLYAQAARNALDAGFDGVELHCANGYLVNQFISAHTNQRD
XenC_TNT11      NALQPDGAAPIAPSAIQAEQAKAFIATGPGVGDLVQPPVPRALTTLEVKELVGHYAQAARNALDAGFDGVEIHAANGYLVNQFISAHANQRD
                .:***.* **:*****:*::.*.** ****.* *.:*..****:* ******  *****************:*.*************:****

XenC_JLR11      LAVAGVVGKERMGVRFAPLFATTDEDRVYLGLVEEDPHQTYIEAVKILEEVGIAYLSLAEADWENAPELPETFREDVRKSFSGKILYAGKYT
XenC_KT2440     LAVAGVVGKERMGVRFAPLFATTDEDRVYLGLVEEDPHQTYIEAVKILEEVGIAYLSLAEADWENAPELPETFREDVRKSFSGKILYAGKYT
XenC_TNT11      EAVSKVVGPERLGVRFSPLFSGTDQDRVYIGLVEDDPHHTYLEAIKVLQAAGIAYVSIAEADWDNAPDLPDTFRQAVRDTFQGRIIYAGRYT
                 **: *** **:****:***: **:****:****:***:**:**:*:*: .****:*:*****:***:**:***: **.:*.*:*:***:**

XenC_JLR11      GRPFIANPDLPARIANNWPLNPVDPSSMYGGTDKGYTDYPTYKS   374
XenC_KT2440     GRPFIANPDLPARIANNWPLNPVDPSSMYGGTDKGYTDYPTYKS
XenC_TNT11      GRPFIANPDLPQRMFNGWPLNPLRAEGMYGGNAVGYIDYPVHAE
                *********** *: *.*****: ...****.  ** ***.: .
```

(d)

```
_KT2440         MSLLLEPYTLRQLTLPNRIAVSPMCQYSAIDGLANDWHLVHLGCRAVGGAGLVISEAVAVTADGRITAEDLGLWNDDQIVPLQRITRFITAQ
_TNT11          MSQLLEPYTLRQLTLLNRIAVSPMCQYSSVDGLANDWHLVHLGSRAVGGAGLIFTEATAITADGRITAEDLGLWNDAQIAPLQRITRFIA--
XenE_TNT19      MSLLLEPYTLRQLTLLNRIAVSPMCQYSSVDGLANDWHLVHLGSRAVGGAGLIFTEATAVTADGRITAEDLGLWNDEQIEPLQPITRFIAAQ
XenE_TNT3       MSLLLEPYTLRQLTLLNRIAVSPMCQYSSVDGLANDWHLVHLGSRAVGGAGLIFTEATAVTADGRITAEDLGLWNDEQIEPLQRITRFIAAQ
                ** ************ ************::*************.********:::**.*:**************** ** *** *****:

XenE_KT2440     RPWLGKQGSVKLEDGGWQPVGPSKIAFDPQHTPPRELSKDEIQDVIDAFVASTERALKAGFKVVEIHAAHGYLLHQFLSPLSNQRRDEYGSC
XenE_TNT11      RPWIGKHGSVKPEDGGWVPVGPSPIAFDPNHTQPRPLDDAGIQQVIADFVAAAKRALTAGFEVVEIHAAHGYLLHQFLSPISNQRQDQYGGT
XenE_TNT19      RPWLGKHGSVKVEDGGWVPVGPSPIAFDPQHTQPTQLDEGAIADIIQAFVDSAKRALVAGFKVVEVHAAHGYLLHQFLSPLSNQRRDQYGGS
XenE_TNT3       RPWLGKHGSVKPEDGGWVPVGPSPIAFDPQHTPPTQLDEGAIAEVIQAFVDSAKRALTAGFKVVEVHAAHGYLLHQFLSPLSNQRRDQYGGS
                ***:**:**** ***** ***** *****:** *  *..  * ::*  ** :::*** ***:***:*************:****:*:**.

XenE_KT2440     WPAELPLFVRVSATDWVEDGWNPDETVELARRLRVLGVDLIDVSSGGTSVNAEIPTGPGYQTRFAERVRKEAEIATGTVGMITEPAQAEHIL
XenE_TNT11      WPQDLPLFVRVSATDWVEDGWNPDETVELARRLKDLGVDLIDVSSGGTAANAEIPIGPGYQTRFAERVRKESGIATGTVGMITEPAQAEHIL
XenE_TNT19      WPEELPLFVRVSATDWVEDGWNPDETVELARRLQTLGVDLIDVSSGGTAVNAEIPIGPGYQTRFAERVRKESGIATGTVGMITEPAQAEHIL
XenE_TNT3       WPEELPVFVRVSATDWVEDGWNPDETVELARRLKALGVDLIDVSSGGTAVNAEIPTGPGYQTRFAERVRKESGIATGTVGMITEPAQAEHIL
                ** :**:************************: *************:.***** ***************: ******************

XenE_KT2440     YWPLHADDDLGGNKATWPAQYQRATSRANPIHESDLRD   368
XenE_TNT11      YWPLHADDDLGGRKATWPAQYQRATHRDQPIHESDLRD
XenE_TNT19      YWPLHADDDLGGRKAVWPAQYQRATHRDQPIHEADLRD
XenE_TNT3       YWPLHADDDLGGRKAIWPAQYQRATHRDQPIHESDLRE
                ************.** ********* * :****:***:
```

Figure S12. Multiple sequence alignment of putative xenobiotic reductases in some pseudomonads. **(a)** XenA from TNT11, *P. putida* II-B (GenBank: AAF02538.1), *P. putida* KT2440 (GenBank: AAN66878.1), and *P. putida* 86 (PDB: 3L5L); **(b)** XenB from TNT isolates, *P. putida* KT2440 (GenBank: AAN66545.1), and *P. fluorescens* I-C (GenBank: AAF02539.1); **(c)** XenC from TNT11 isolate, *P. putida* strain KT2440 (GenBank: AAN68098.1), and JLR11 (protein sequence from genome re-annotation); **(d)** XenE from TNT isolates and *P. putida* KT2440 (GenBank: AAN67100. Residues involved in FMN, NADPH, substrate, and FMN/NADPH binding are shown in red, green, gray, and orange. Conserved residues are shown in blue. Flavin mononucleotide (FMN), Nicotinamide adenine dinucleotide phosphate (NADPH), Protein Data Bank (PDB).

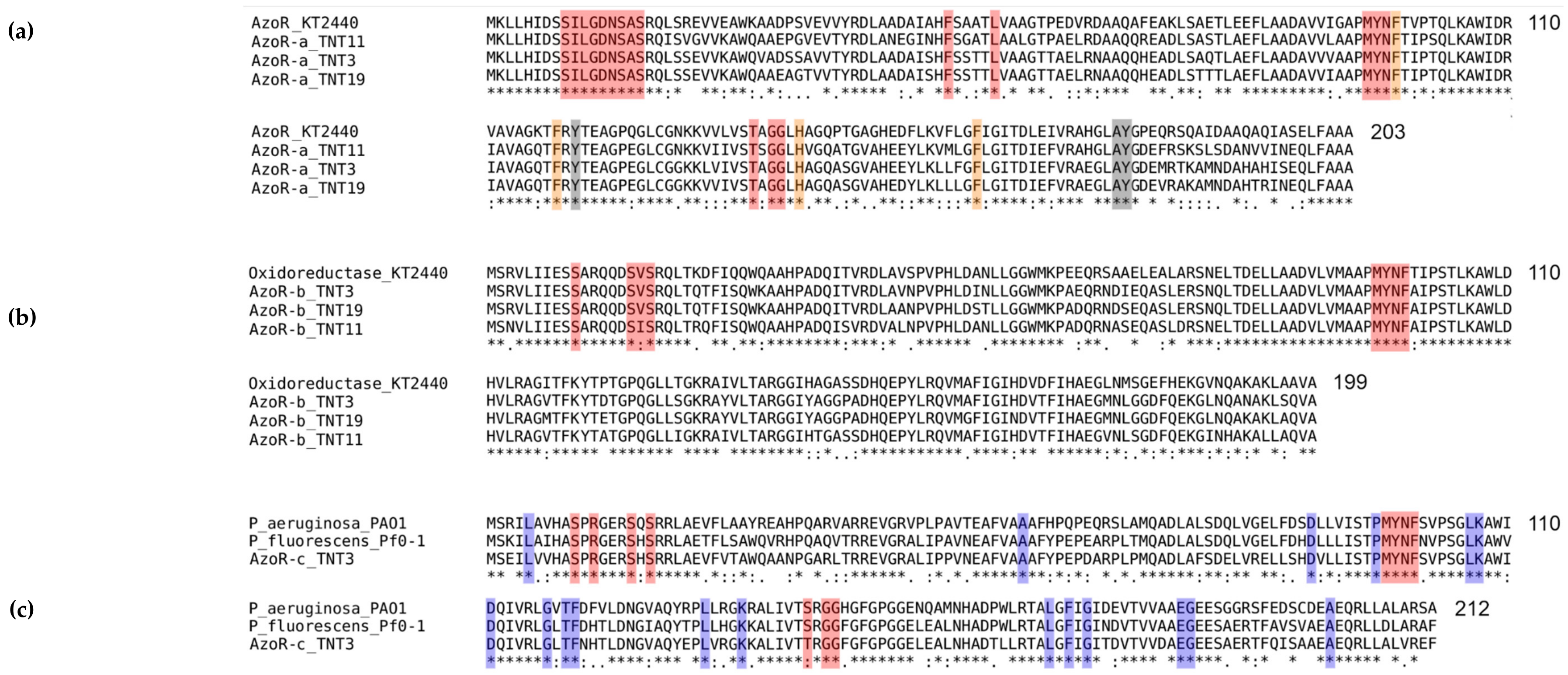

Figure S13. Multiple sequence alignment of putative azoreductases in some pseudomonads. **(a)** AzoR-a from TNT isolates and MULTISPECIES: FMN-dependent NADH-azoreductase from *P. putida* KT2440 (GenBank: WP_010953771.1); **(b)** AzoR-b from TNT isolates and FMN-dependent NADH:quinone oxidoreductase 2 from *P. putida* KT2440 (UniProtKB: Q88EC8); **(c)** AzoR-c from TNT3 isolate, FMN-dependent NADH-azoreductase 1 from *P. aeruginosa* PAO1 (GenBank: AAG04174.1) and FMN-dependent NADH:quinone oxidoreductase 1 from *P. fluorescens* Pf0-1 (UniProtKB: Q3KIU3). Residues involved in FMN, substrate, and FMN/NADPH binding are shown in red, gray, and orange. Conserved residues are shown in blue. Flavin mononucleotide (FMN), Nicotinamide adenine dinucleotide phosphate (NADPH), Protein Data Bank (PDB).

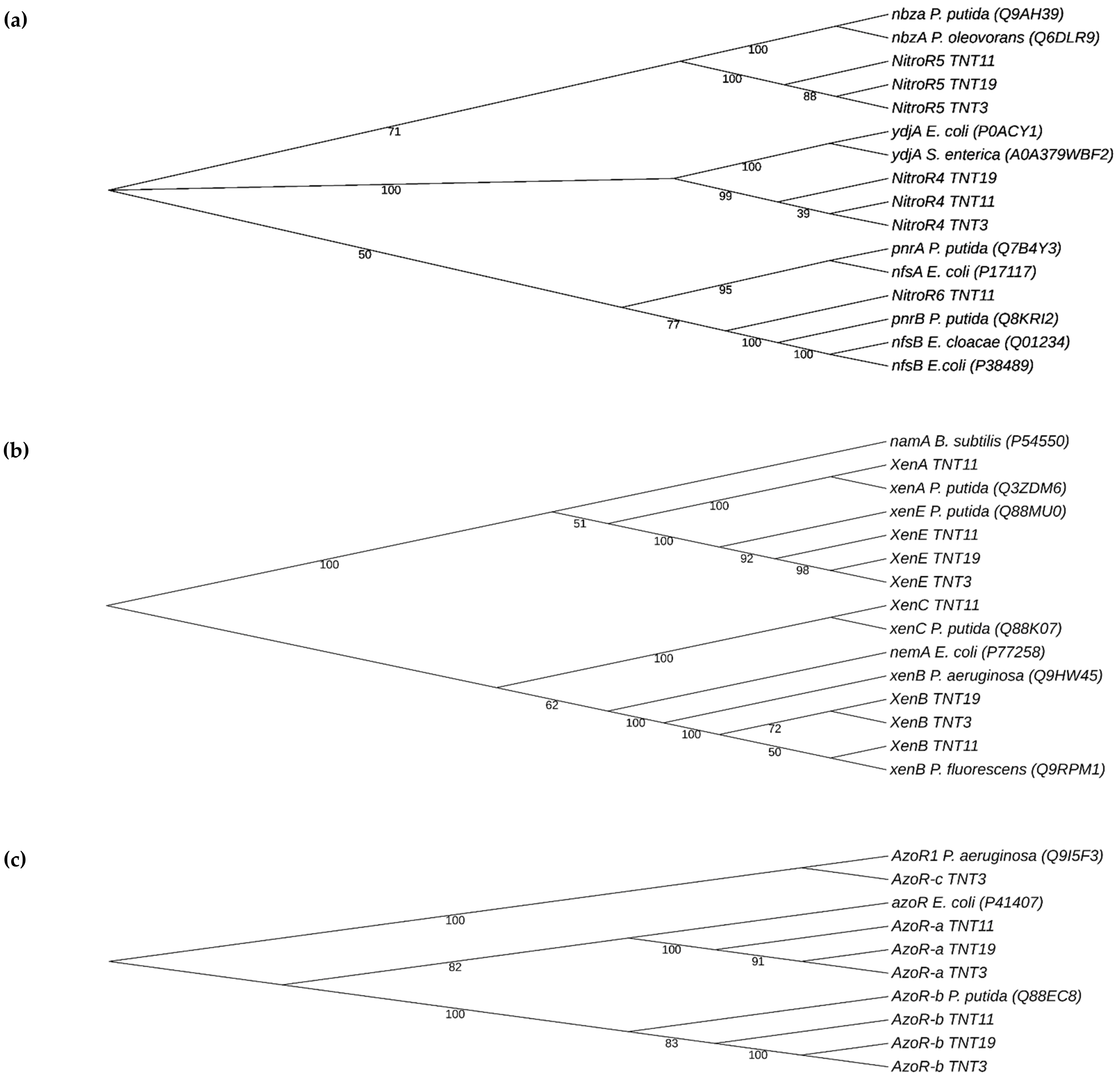

Figure S14. Neighbor-Joining (NJ) cladogram depicting phylogenetic relationships among TNT-degrading enzymes. **(a)** Nitroreductases, **(b)** Old Yellow Enzymes, and **(c)** Azoreductases from TNT isolates, using Jones-Taylor-Thornton (JTT) substitution model. Numbers at the branches indicate bootstrap support values (100 bootstrap samples). Sequences were aligned with MAFFT (L-INS-i). UniProtKB accessions for characterized enzymes are shown in parentheses. Nitrobenzene reductase (nbza), Nitroreductase (NitroR), Xenobiotic reductase (xen), *N*-ethylmaleimide reductase (nemA), Azoreductase (AzoR).
