# Supplementary material for: Comparative Genomic Analysis of Antarctic Pseudomonas Isolates with 2,4,6-Trinitrotoluene Transformation Capabilities Reveals Their Unique Features for Xenobiotics Degradation
Source: Genes (Basel). 2022 Jul 28;13(8):1354. doi: 10.3390/genes13081354 (PMC9407559; doi:10.3390/genes13081354)
Supplement: Supplementary file 1 [file genes-13-01354-s001.zip › supplementary_tables2507.pdf]

Table S1. Characteristics of the pseudomonads used for comparative genomic analysis in this study.

Bacterium	Selection criterion	Isolation site	Characteristics	Genome size (Mbp)	G+C content (%)	Genome status	GenBank assembly accession	Reference
Pseudomonas sp. TNT3	TNT degradation	Deception Island (Antarctica)	TNT consumption	6.46	58.6	Draft	GCA_010095445.1	This study [29]
Pseudomonas sp. TNT11	TNT degradation	Deception Island (Antarctica)	TNT consumption	5.86	60.4	Draft	Unavailable	This study [29]
Pseudomonas sp. TNT19	TNT degradation	Deception Island (Antarctica)	TNT consumption	6.45	58.6	Draft	Unavailable	This study [29]
P. putida JLR11	TNT degradation	Wastewater treatment plant (Spain)	TNT degradation. Use of TNT as a sole nitrogen source.	6.10	61.6	Draft	GCA_001183585.1	[109,110]
P. putida KT2440	TNT degradation. Non-pathogenic bacterium.	Derivative of *P. putida* mt-2 (an aromatic hydrocarbon-degrader)	TNT degradation. High resistance to oxidative stress. Model bacterium and non-pathogenic.	6.18	61.5	Complete	GCA_000007565.2	[30,89]

Bacterium	Selection criterion	Isolation site	Characteristics	Genome size (Mbp)	G+C content (%)	Genome status	GenBank assembly accession	Reference
P. aeruginosa PFL-P1	Aromatic compounds degradation.	Polycyclic Aromatic Hydrocarbons (PAHs)-contaminated marine site (India).	Phenanthrene degradation	6.33	66.5	Draft	GCA_013309705.1	[111]
P. frederiksbergensis AS1	Closely related to TNT isolates. Aromatic compounds degradation.	Arsenic-contaminated site (South Korea)	Growth on naphthalene and various aromatic compounds	6.21	58.9	Complete	GCA_001952935.1	[71,112]
P. veronii 1YdBTEX2	Closely related to TNT isolates. Aromatic compounds degradation.	Benzene-contaminated site (Czech Republic)	Benzene and toluene degradation	6.68	60.6	Draft	GCA_000350565.1	[67]
P. veronii Pvy	Closely related to TNT isolates. Aromatic compounds degradation.	Sediment from a lagoon near an oil refinery (Romania)	Dibenzofuran degradation and utilization of various aromatic compounds.	7.31	60.7	Complete	GCA_004919535.4	[68]

Pseudomonas sp. GC01	Antarctic origin	Soil sample from Deception Island (Antarctica)	CdS nanoparticles biosynthesis. High resistance to cadmium. Psychrotolerant bacterium	4.71	58.6	Draft	GCA_013002835.1	[113]
Pseudomonas sp. MPC6	Antarctic origin	Soil sample from Deception Island (Antarctica)	Natural Polyhydroxyalkanoates (PHAs) production. High resistance to heavy metals.	7.22	60.0	Complete	GCA_006094435.1	[28]
P. deceptionensis LMG 25555	Antarctic origin	Marine sediment in Deception Island (Antarctica)	Psychrotolerant bacterium	5.10	58.5	Draft	GCA_900106095.1	[114]
P. antarctica PAMC 27494	Antarctic origin	Freshwater sample from Antarctica	Psychrophile with antimicrobial activity	6.61	59.7	Complete	GCA_001647715.1	[115]
P. fildesensis KG01	Antarctic origin	Soil sample from King George Island (Antarctica)	Psychrotolerant bacterium	6.31	60.1	Draft	GCA_001050345.1	[116]
P. extremaustralis 14-3b	Antarctic origin	Natural derivative of *P. extremaustralis* 14-3 (isolated from a temporary pond in Antarctica)	Polyhydroxybutyrate (PHB) production. High resistance to stress.	6.59	60.7	Draft	GCA_000242115.2	[117]
P. mandelii PD30	Closely related to TNT isolates	Agricultural soil samples from potato fields (Canada)	Denitrifying bacterium	6.69	59.0	Draft	GCA_000690555.2	[70]

P. mandelii DSM 17967	Closely related to TNT isolates	Natural mineral waters (France)	Not reported	6.99	59.3	Draft	GCA_007858265.1	[118]
P. frederiksbergensis ERDD5:01	Closely related to TNT isolates	Sedimentary soil of a glacier stream (Sikkim Himalaya)	High tolerance to cold and UV-C radiation	6.12	55.0	Complete	GCA_001874645.1	[119]
P. fluorescens F113	Closely related to TNT isolates	Sugar-beet rhizosphere	Plant growth-promoting rhizobacterium.	6.85	60.8	Complete	GCA_000237065.1	[120]
P. fluorescens ATCC 13525	Closely related to TNT isolates. Non-pathogenic bacterium.	Pre-filter tanks (England)	Non-pathogenic	6.51	60.0	Complete	GCA_900215245.1	[121]
P. aeruginosa PAO1	Human pathogen	Derivative of *P. aeruginosa* PAO (isolated from a wound Australia)	Human pathogenic bacterium. Chloramphenicol resistance.	6.26	66.6	Complete	GCA_000006765.1	[85]

**Bacterium**	**Selection criterion**	**Isolation site**	**Characteristics**	**Genome size (Mbp)**	**G+C content (%)**	**Genome status**	**GenBank assembly accession**	**Reference**
P. aeruginosa UCBPP-PA14	Human pathogen	Human burn patient	High virulence. Pathogenic for human, mice, and *Arabidopsis thaliana*.	6.54	66.3	Complete	GCA_000014625.1	[86,122]

P. syringae pv. *syringae* strain B301D	Plant pathogen	Diseased flower of the common pear (England)	Pathogenic for cherry and apricot	6.10	59.2	Complete	GCA_000988485.1	[87]
P. syringae pv. *syringae* strain HS191	Plant pathogen	Diseased proso millet (Australia)	Pathogenic for maize	6.00	59.0	Complete	GCA_000988395.1	[87]

Table S2. Enzymes encoded by genes of the "Trinitrotoluene degradation" pathway found by PATRIC/RASTtk in TNT isolates genomes.

	**Isolate**		
**No.**	**TNT3**	**TNT11**	**TNT19**
1	Probable *N*-methylproline demethylase (EC 1.-.-.-)	Beta-ketoadipate enol-lactone hydrolase (EC 3.1.1.24)	Probable N-methylproline demethylase (EC 1.-.-.-)
2	Probable VANILLIN dehydrogenase oxidoreductase protein (EC 1.-.-.-)	Aldehyde dehydrogenase (EC 1.2.1.3)	Probable oxidoreductase ordL (EC 1.-.-.-)
3	L-ornithine 5-monooxygenase (EC 1.13.12.-)	Quino(hemo)protein alcohol dehydrogenase, PQQ-dependent (EC 1.1.2.8)	
4	Probable oxidoreductase ordL (EC 1.-.-.-)	Alkaline phosphatase (EC 3.1.3.1)	
5		Allophanate hydrolase 2 subunit 1 (EC 3.5.1.54)	
6		Isonitrile hydratase (EC 4.2.1.103)	
7		Probable VANILLIN dehydrogenase oxidoreductase protein (EC 1.-.-.-)	
8		Glutathione *S*-transferase (EC 2.5.1.18)	
9		Homogentisate 1,2-dioxygenase (EC 1.13.11.5)	

Table S3. The pangenome of the 24 pseudomonads used in this study and groups of sub-pangenomes constructed with Roary. Group 1: Species with experimentally demonstrated TNT transformation capability whose genomes are publicly available; Group 2: Species with experimentally demonstrated xenobiotics degradation capability, including aromatics (other than TNT); Group 3: Species of Antarctic origin; Group 4: Species with close phylogenetic relationships to TNT isolates; Group 5: Pathogenic species. All groups include TNT isolates. Categories are considered enriched if their total count is ≥ 10% for COGs. Number of species (#) considering each TNT isolate as a novel species. The following are the descriptions of COG categories enriched: (C) Energy production and conversion; (E) Amino acid transport and metabolism; (J) Translation, ribosomal structure and biogenesis; (K) Transcription; (S) Function unknown.

**Feature**	**Pangenome**						
	**Full set**	**Group 1**	**Group 2**	**Group 3**	**Group 4**	**Group 5**	**TNT isolates**
Genomes (*n*)	24	5	7	9	12	7	3
#species	11-16	4	6	7-9	7	5	3
Pangenome size (genes)	30,253	7,896	8,211	7,334	10,797	11,825	2,369
Average % of genes in the core	0.1	1.4	0.2	4.6	5.0	0.2	25.9
Total core genes (No.)	29	112	17	338	541	19	613
Hard core genes	27	112	17	338	541	19	613
Soft core genes	2	0	0	0	0	0	0

Total accessory genes (No.)	30,224	7,784	8,194	6,996	10,256	11,806	1,756
Shell genes	4,703	7,784	7,149	5,782	8,813	11,329	1,756
Cloud genes	25,521	0	1,045	1,214	1,443	477	0
COGs enriched in core	J,C,K	J,K,C	J,K	J,K,E,C	J,E,K	J,K	J,K,E,C
COGs enriched in accessory	S	S	S	S,E	S	S	S,E

Table S4. Prophage regions found in the genomes of TNT-transforming bacteria.

Bacterium	Region	Completeness	Region length (Kb)	Region position	GC content (%)	Total proteins	Phage hit proteins	Hypothetical proteins	Phage-related keyword	Most common phage (GenBank accession code)
TNT3	1	Intact	57.3	Node 8 (99815-157166)	55.5	62	43	19	Integrase, tail, terminase, portal, capsid, head	*Burkholderia* phage Bcep176 (NC_007497)
TNT11	1	Intact	22.0	Node 2 (14856-36863)	58.4	35	22	13	Tail, virion, capsid, head, portal, terminase	*Pseudomonas* phage YMC11/02/R656 (NC_028657)
	2	Intact	36.1	Node 12 (1528-37637)	58.5	52	41	11	Head, plate, capsid, portal, terminase, tail, recombinase	*Edwardsiella* phage PEi21 (NC_021342)
	3	Intact	33.2	Node 14 (1821-35114)	58.4	47	37	10	Tail, plate, head, portal, terminase	*Salmonella* phage SEN34 (NC_028699)
	4	Incomplete	21.8	Node 3 (3731-25586)	58.8	15	8	7	Integrase, tail	*Escherichia* phage 500465-1 (NC_049342)
	5	Incomplete	23.6	Node 5 (719-24328)	57.3	19	12	7	Integrase	*Pseudomonas* phage phiPSA1 (NC_024365)

**Bacterium**	**Region**	**Completeness**	**Region length (Kb)**	**Region position**	**GC content (%)**	**Total proteins**	**Phage hit proteins**	**Hypothetical proteins**	**Phage-related keyword**	**Most common phage (GenBank accession code)**
TNT19	1	Intact	35.5	Node 3 (224212-259714)	56.9	37	32	5	Lysin	*Pseudomonas* phage phi3 (NC_030940)
	2	Intact	35.5	Node 9 (3304-38831)	56.7	45	38	7	Tail, plate, head, virion, portal, terminase	*Pseudomonas* phage B3 (NC_006548)
	3	Incomplete	5.9	Node 1 (205025-210936)	55.4	11	6	5	Transposase,tail	Stx2-converting phage 1717 (NC_011357)
	4	Incomplete	17.5	Node 14 (40091-57592)	59.0	17	8	9	Tail,integrase	*Escherichia* phage 500465-1 (NC_049342)
TNT19	5	Incomplete	23.4	Node 28 (46772-70212)	56.5	23	17	6	Integrase	*Pseudomonas* phage phi3 (NC_030940)
JLR11	1	Intact	39.1	NZ_LDJF01000018.1 (32818-72004)	58.4	45	29	16	Lysin,tail	*Ralstonia* phage DU_RP_I (NC_047888)
	2	Intact	55.9	NZ_LDJF01000024.1 (200654-256635)	59.4	77	51	26	Tail,plate,capsid,terminase,integrase	*Salmonella* phage 118970_sal3 (NC_031940)

	3	Intact	45.2	NZ_LDJF01000008.1 (298761-344004)	61.3	54	42	12	Integrase,capsid,tail,terminase,head	*Pseudomonas* phage PMG1 (NC_016765)
	4	Incomplete	27.6	NZ_LDJF01000018.1 (276978-304651)	57.5	9	6	3	Tail,integrase	*Burkholderia* phage phi1026b (NC_005284)
	5	Incomplete	13.7	NZ_LDJF01000018.1 (801949-815701)	60.2	15	9	6	Tail,transposase	*Sinorhizobium* phage phiN3 (NC_028945)
	6	Incomplete	14.0	NZ_LDJF01000018.1 (858902-872911)	59.2	12	8	4	Tail	*Burkholderia* phage Bcep176 (NC_007497)
	7	Incomplete	8.9	NZ_LDJF01000024.1 (303136-312117)	57.9	11	8	3	Tail,transposase	*Escherichia* phage SH2026Stx1 (NC_049919)
KT2440	1	Intact		1734867-1780110	61.3	56	41	15	Integrase,capsid,tail, terminase,head	*Pseudomonas* phage PMG1 (NC_016765)
	2	Intact		2586636-2625822	58.4	45	29	16	Lysin,tail	*Ralstonia* phage DU_RP_I (NC_047888)

Bacterium	Region	Completeness	Region length (Kb)	Region position	GC content (%)	Total proteins	Phage hit proteins	Hypothetical proteins	Phage-related keyword	Most common phage (GenBank accession code)
KT2440	3	Intact		3426052-3447795	63	26	21	5	Tail,portal,head,plate	*Vibrio* phage vB_VpaM_MAR (NC_019722)
	4	Intact		4371608-4427499	59.4	73	50	23	Tail,plate,capsid,terminase,integrase	*Salmonella* phage 118970_sal3 (NC_031940)
	5	Incomplete		2831045-2858718	57.5	9	6	3	Tail,integrase	*Xanthomonas* phage Carpasina (NC_047962)
	6	Incomplete		3355957-3369709	60.2	15	9	6	Tail,transposase	Cyanophage S-RIM12 isolate W1_08_0910 (NC_047717)
	7	Incomplete		3412910-3426919	59.2	12	8	4	Tail	*Burkholderia* phage Bcep176 (NC_007497)
	8	Incomplete		3522420-3531285	63.9	9	6	3	Tail,transposase	Stx2-converting phage Stx2a_F451 proviral DNA (NC_049924)

9	Incomplete	4474000-4482981	57.9	11	8	3	Tail,transposase	Stx2-converting phage Stx2a_F451 proviral DNA (NC_049924)

Table S5. Putative TNT-degrading enzymes found in TNT isolates.

Type of enzyme	Enzyme name	Bacterium	Length sequence (residues)	Hypothetical molecular mass (kDa)	Best hit (% identity, enzyme, bacterium, accession number)
Nitroreductase	NitroR4	TNT3	187	20.1	a) 70,2%, NitroR2, *P. putida* strains JLR11 and KT2440<br>b) 84.8%, putative NAD(P)H nitroreductase, *Pseudomonas* sp. MF4836, UniProtKB: A0A1T1HZT3
		TNT11	187	20.0	a) 68,1%, NitroR2, *P. putida* strains JLR11 and KT2440<br>b) 94.1%, putative NAD(P)H nitroreductase, *P. fluorescens* ABAC62, UniProtKB: A0A656YN39
		TNT19	188	20.2	a) 67,6%, NitroR2, *P. putida* strains JLR11 and KT2440<br>b) 82.4%, putative NAD(P)H nitroreductase, *Pseudomonas* sp. MF4836, UniProtKB: A0A1T1HZT3
	NitroR5	TNT3	197	21.9	91.4%, nitroreductase family protein, *Pseudomonas* sp. FW507-12TSA, UniProtKB: A0A2K4IRJ3
		TNT11	197	22.0	87.3%, nitroreductase family protein, *P. syringae* pv. *tomato* ATCC BAA-871, UniProtKB: Q87Y84
		TNT19	197	21.9	91.4%, nitroreductase (*P. putida* CSV86, UniProtKB: L1M0Z3
	NitroR6	TNT11	204	21.7	82.8%, nitroreductase, *P. mucidolens*, UniProtKB: A0A1H2MQ87
	XenA	TNT11	363	39.6	91.74%, XenA, *P. putida* II-B, GenBank: AAF02538.1

Xenobiotic reductase	XenB	TNT3	349	37.6	91.7%, XenB, *P. fluorescens* I-C, GenBank: AAF02539.1
		TNT11	351	37.5	93.1%, XenB, *P. fluorescens* I-C, GenBank: AAF02539.1
		TNT19	349	37.6	94.3%, XenB, *P. fluorescens* I-C, GenBank: AAF02539.1
	XenC	TNT11	374	40.7	72.7%, XenC, KT2440, GenBank: AAN68098.1
	XenE	TNT3	368	40.4	87.8%, XenE, KT2440, GenBank: AAN67100.1
		TNT11	352	38.9	83.5%, XenE, KT2440, GenBank: AAN67100.1
		TNT19	368	40.4	85.3%, XenE, KT2440, GenBank: AAN67100.1

**Type of enzyme**	**Enzyme name**	**Bacterium**	**Length sequence (residues)**	**Hypothetical molecular mass (kDa)**	**Best hit (% identity, enzyme, bacterium, accession number)**
Azoreductase	AzoR (named AzoR-a)	TNT3	203	21.4	72.9%, AzoR, KT2440, GenBank: WP_010953771.1
		TNT11	203	21.5	72.4%, AzoR, KT2440, GenBank: WP_010953771.1
		TNT19	203	21.4	73.4%, AzoR, KT2440, GenBank: WP_010953771.1
		TNT3	199	21.8	82.9%, AzoR2, KT2440, UniProtKB: Q88EC8

AzoR2 (named AzoR-b)	TNT11	199	21.6	84.4%, AzoR2, KT2440, UniProtKB: Q88EC8
	TNT19	199	21.7	81.4%, AzoR2, KT2440, UniProtKB: Q88EC8
AzoR1 (named AzoR-c)	TNT3	212	23.1	72.1%, AzoR1, PAO1, GenBank: AAG04174.1
