# Supplementary figures and images for: Comparative Genomic Analysis of Antarctic Pseudomonas Isolates with 2,4,6-Trinitrotoluene Transformation Capabilities Reveals Their Unique Features for Xenobiotics Degradation
Source: Genes (Basel). 2022 Jul 28;13(8):1354. doi: 10.3390/genes13081354 (PMC9407559; doi:10.3390/genes13081354)

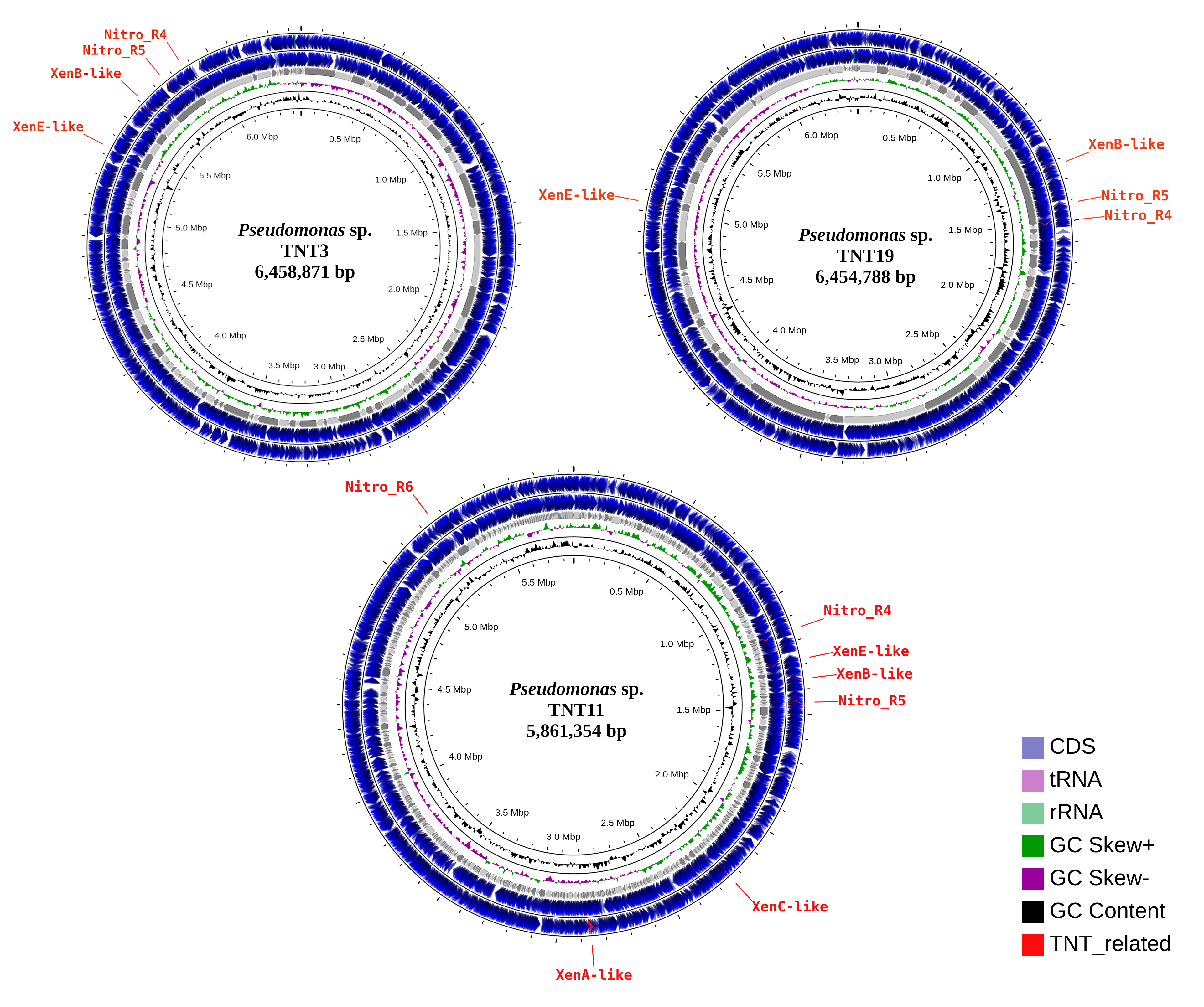

Supplement: Supplementary file 1 [file genes-13-01354-s001.zip › supplementary_figures/fig_S1.png]

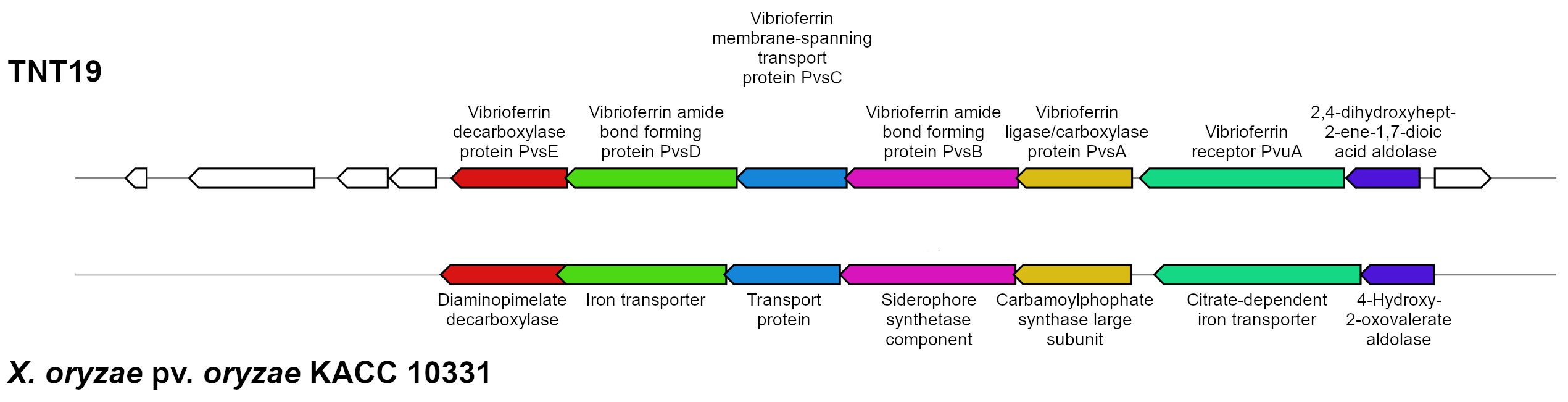

Supplement: Supplementary file 1 [file genes-13-01354-s001.zip › supplementary_figures/fig_S10.png]

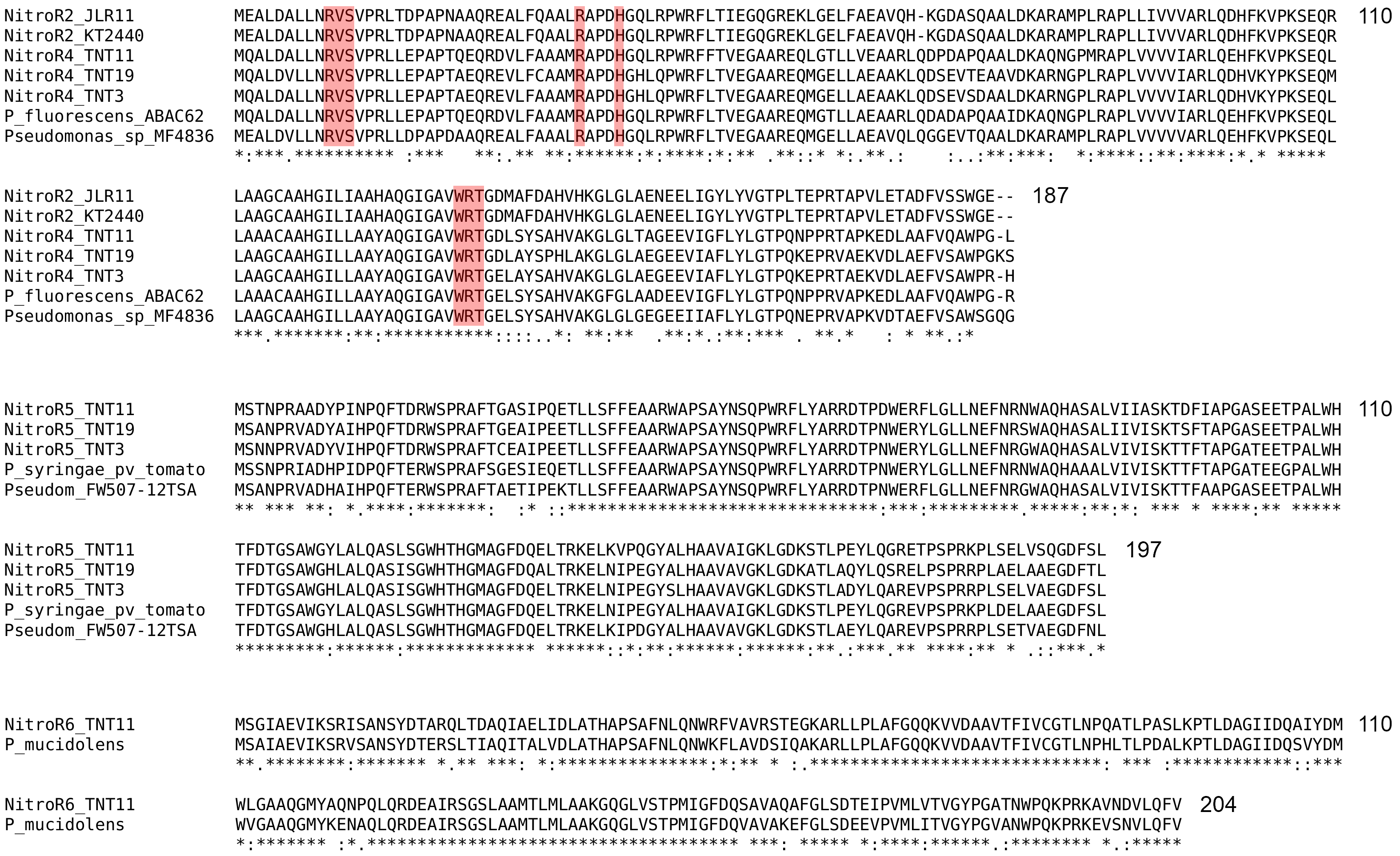

Supplement: Supplementary file 1 [file genes-13-01354-s001.zip › supplementary_figures/fig_S11.png]

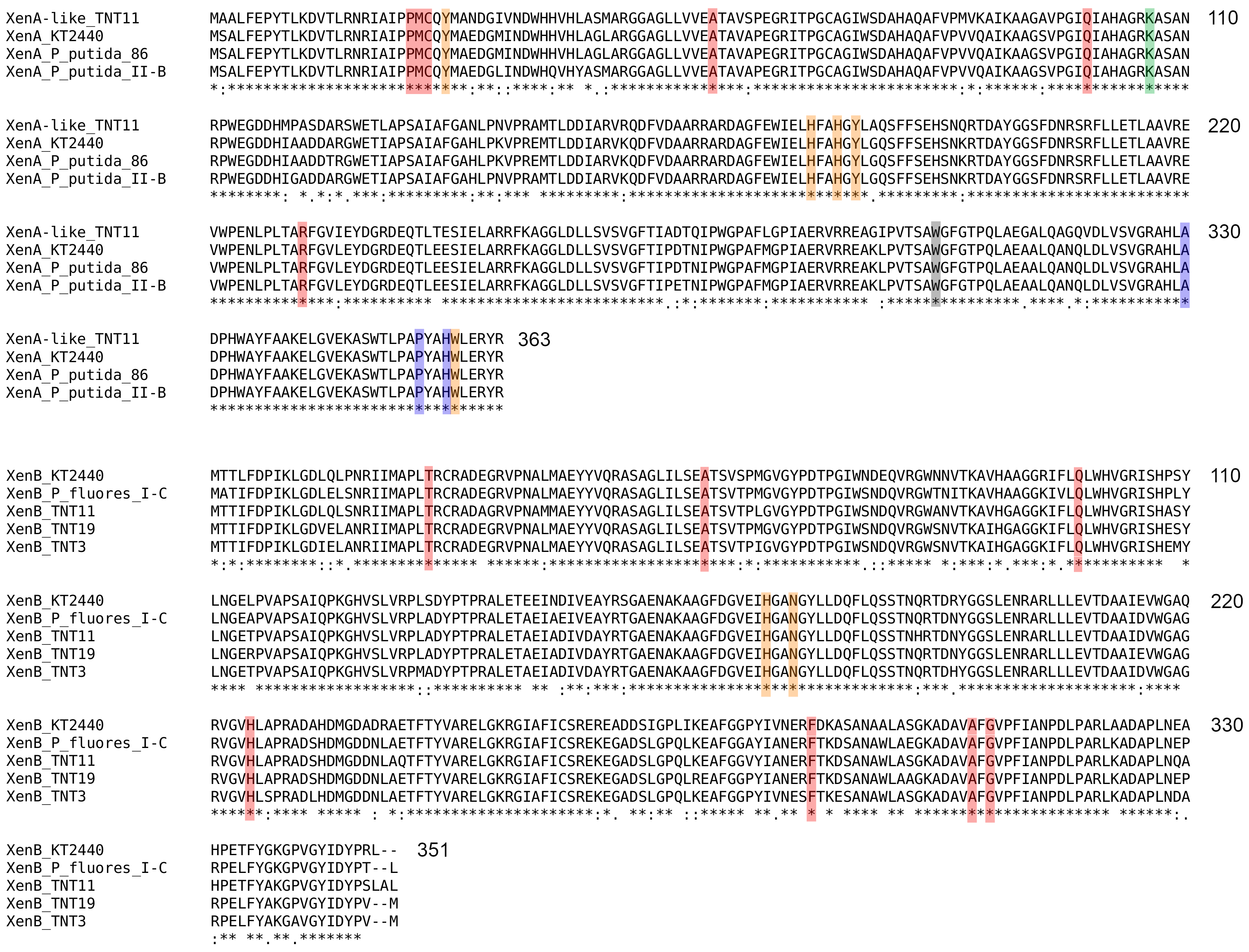

Supplement: Supplementary file 1 [file genes-13-01354-s001.zip › supplementary_figures/fig_S12-1.png]

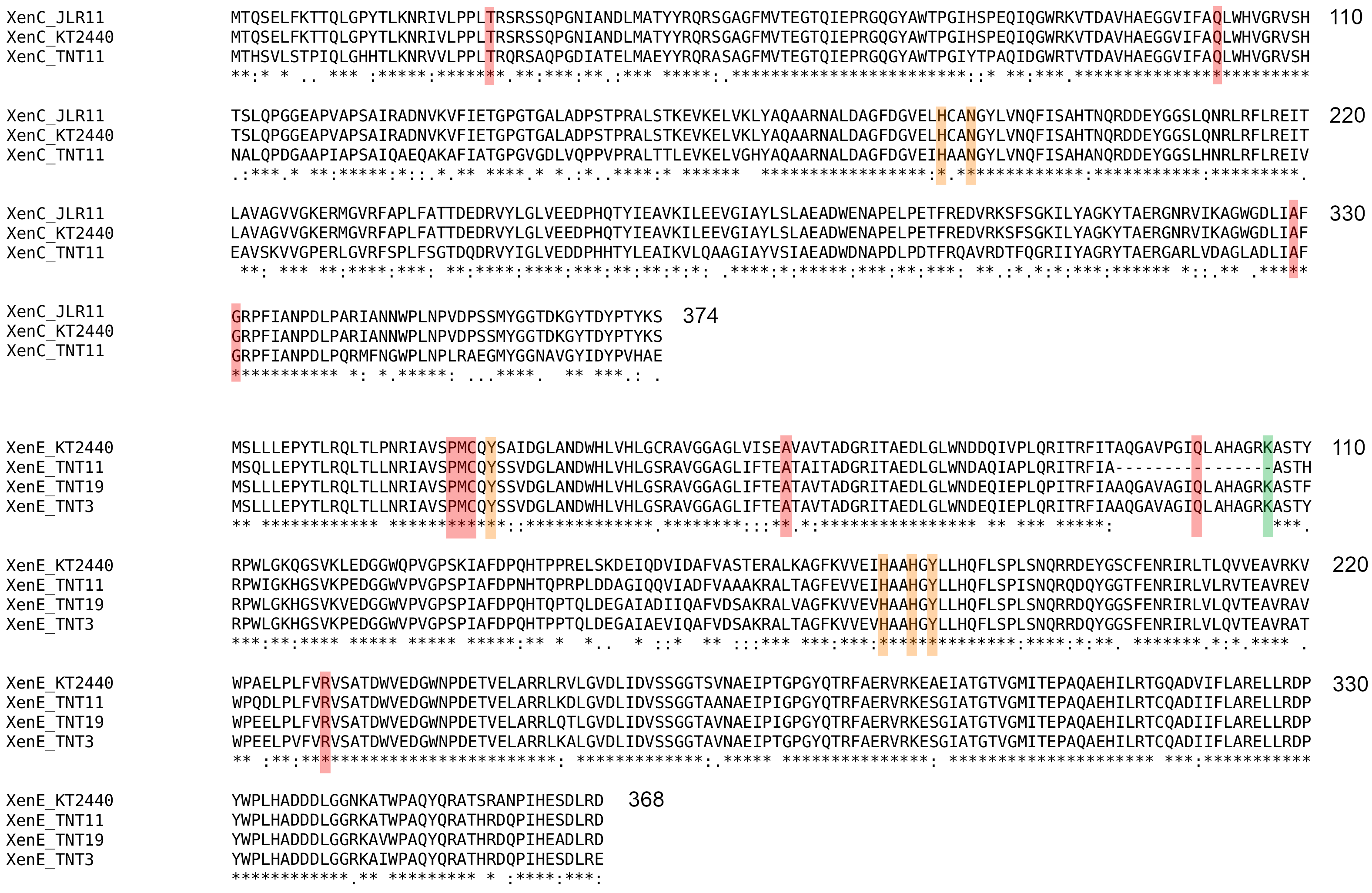

Supplement: Supplementary file 1 [file genes-13-01354-s001.zip › supplementary_figures/fig_S12-2.png]

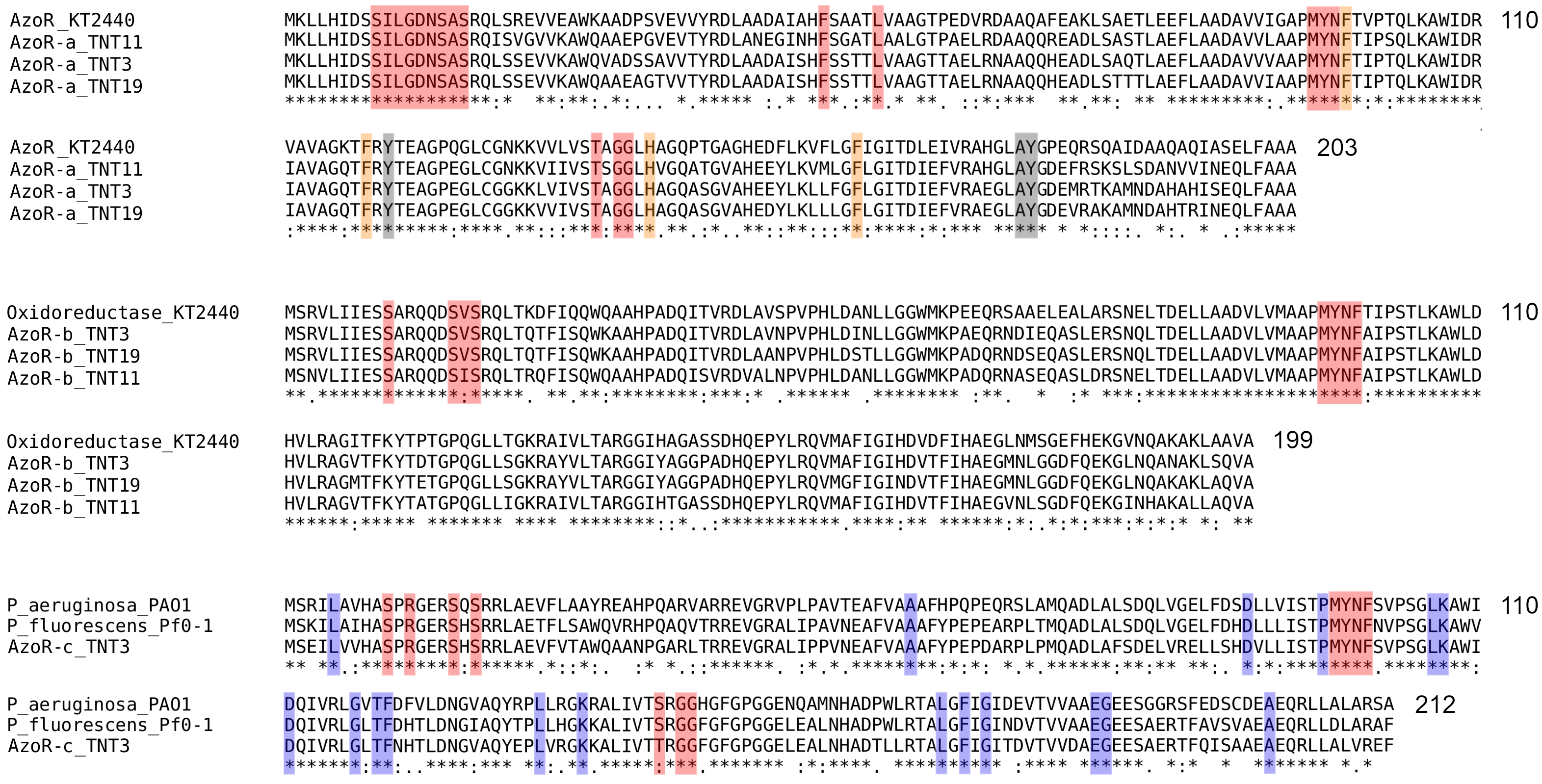

Supplement: Supplementary file 1 [file genes-13-01354-s001.zip › supplementary_figures/fig_S13.png]

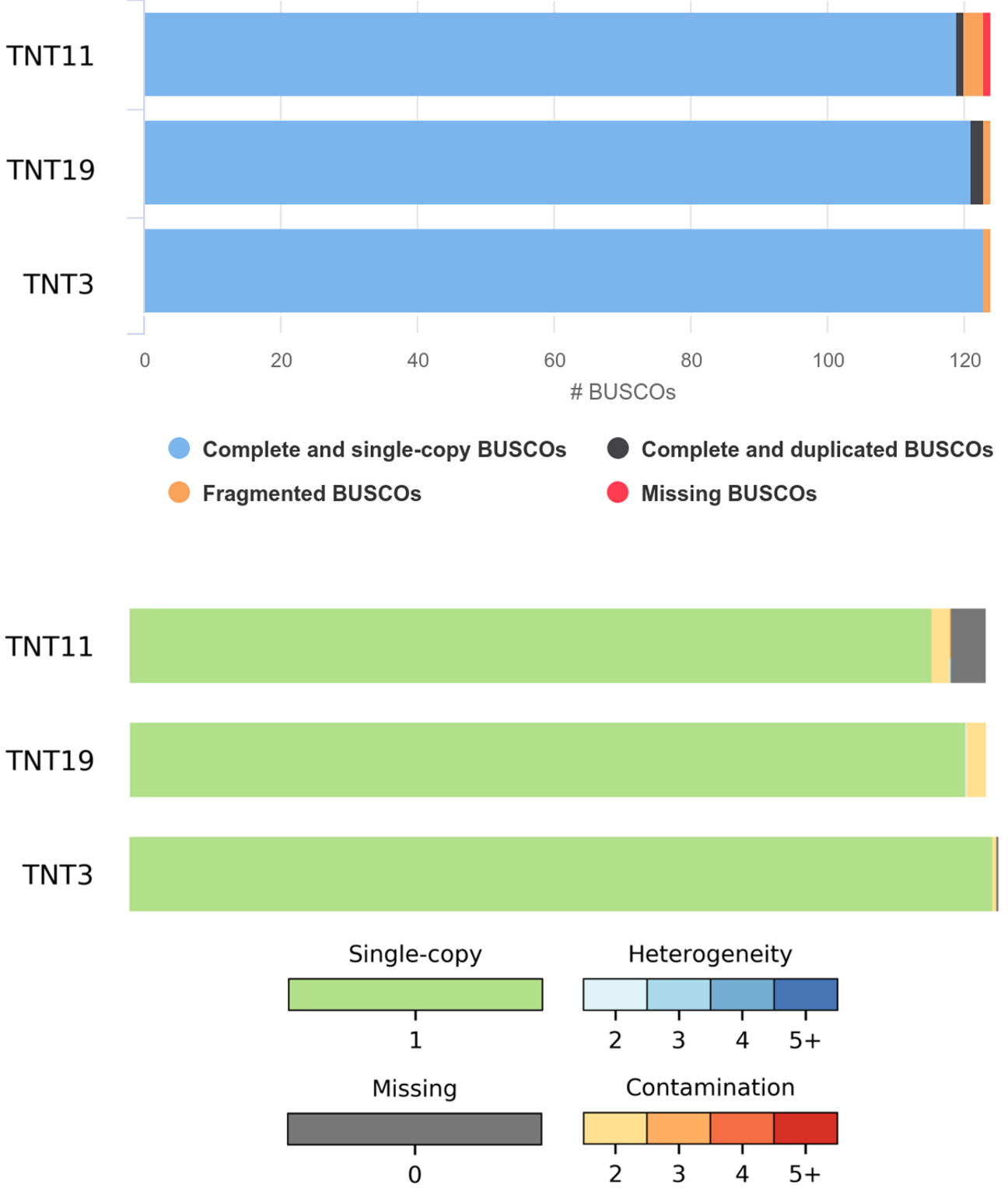

Supplement: Supplementary file 1 [file genes-13-01354-s001.zip › supplementary_figures/fig_S2.png]

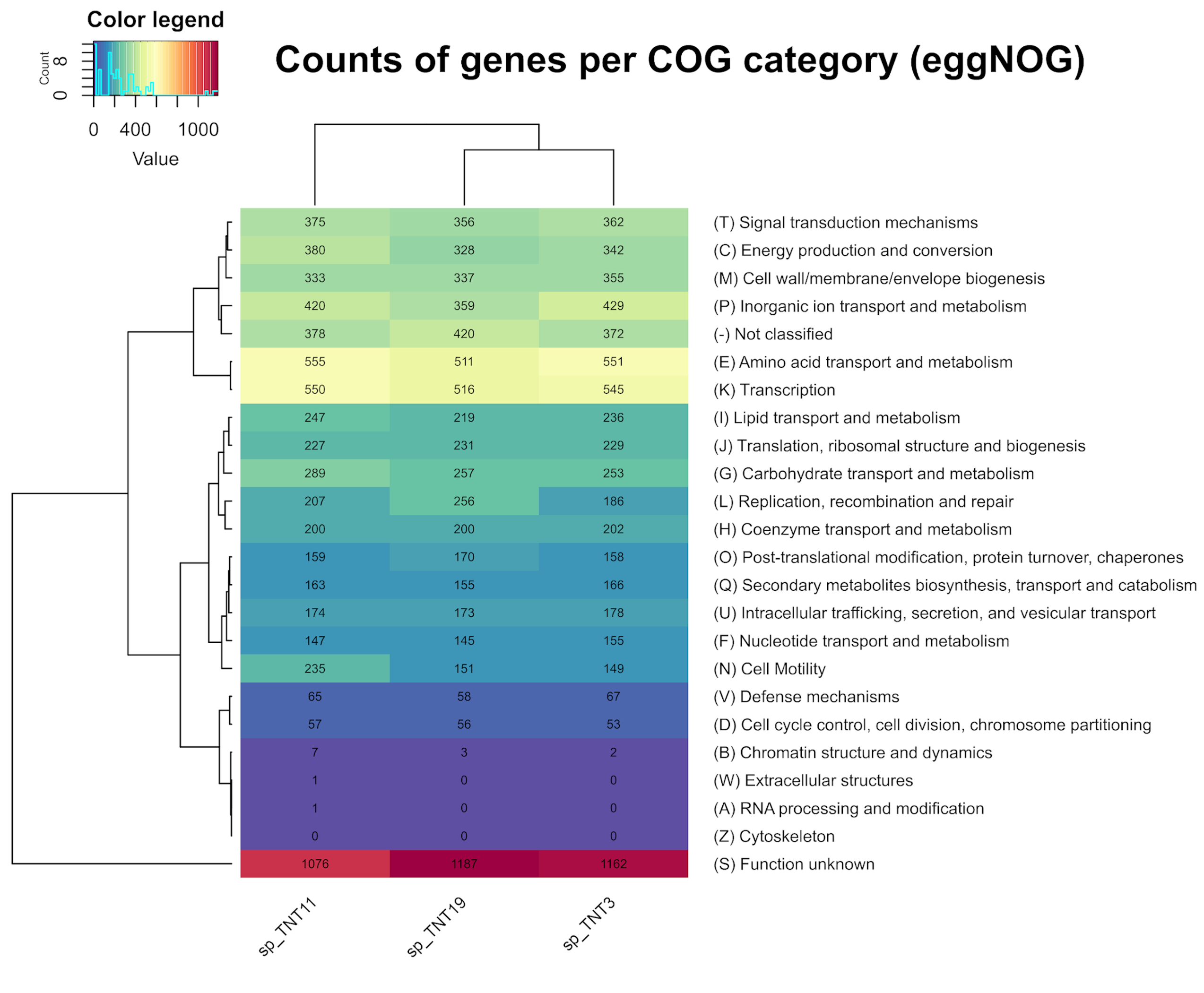

Supplement: Supplementary file 1 [file genes-13-01354-s001.zip › supplementary_figures/fig_S3.png]

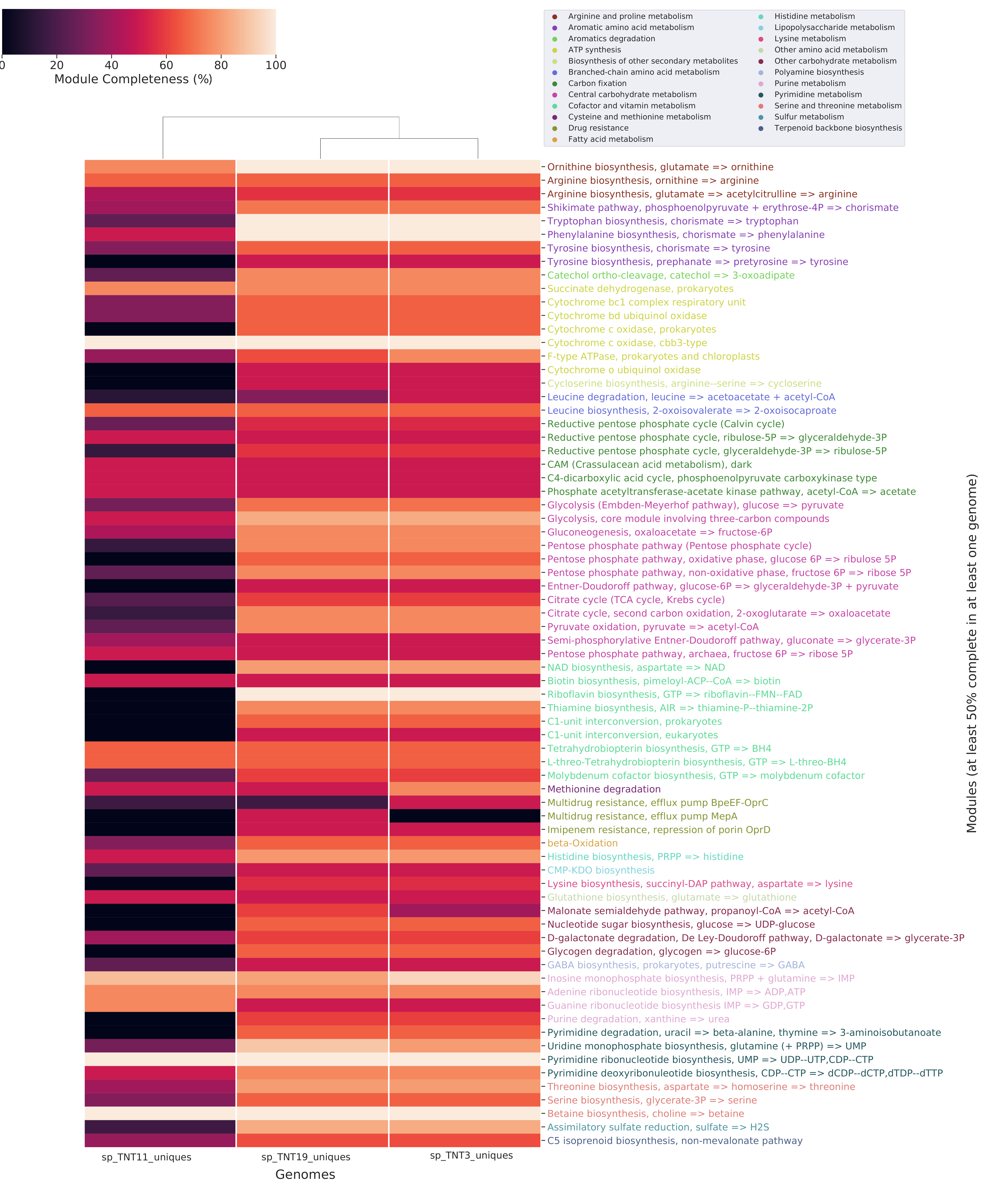

Supplement: Supplementary file 1 [file genes-13-01354-s001.zip › supplementary_figures/fig_S7.png]

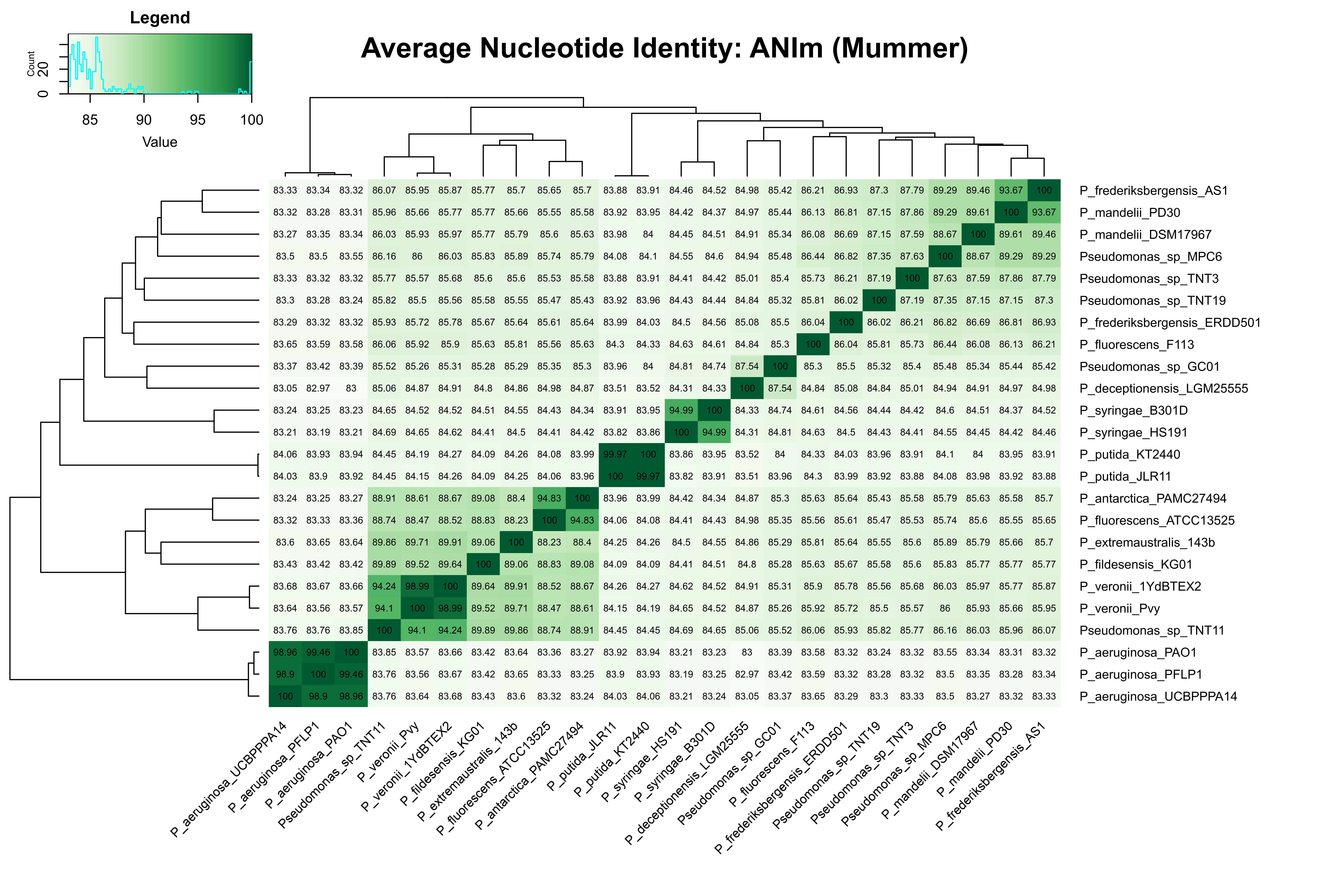

Supplement: Supplementary file 1 [file genes-13-01354-s001.zip › supplementary_figures/fig_S8.png]

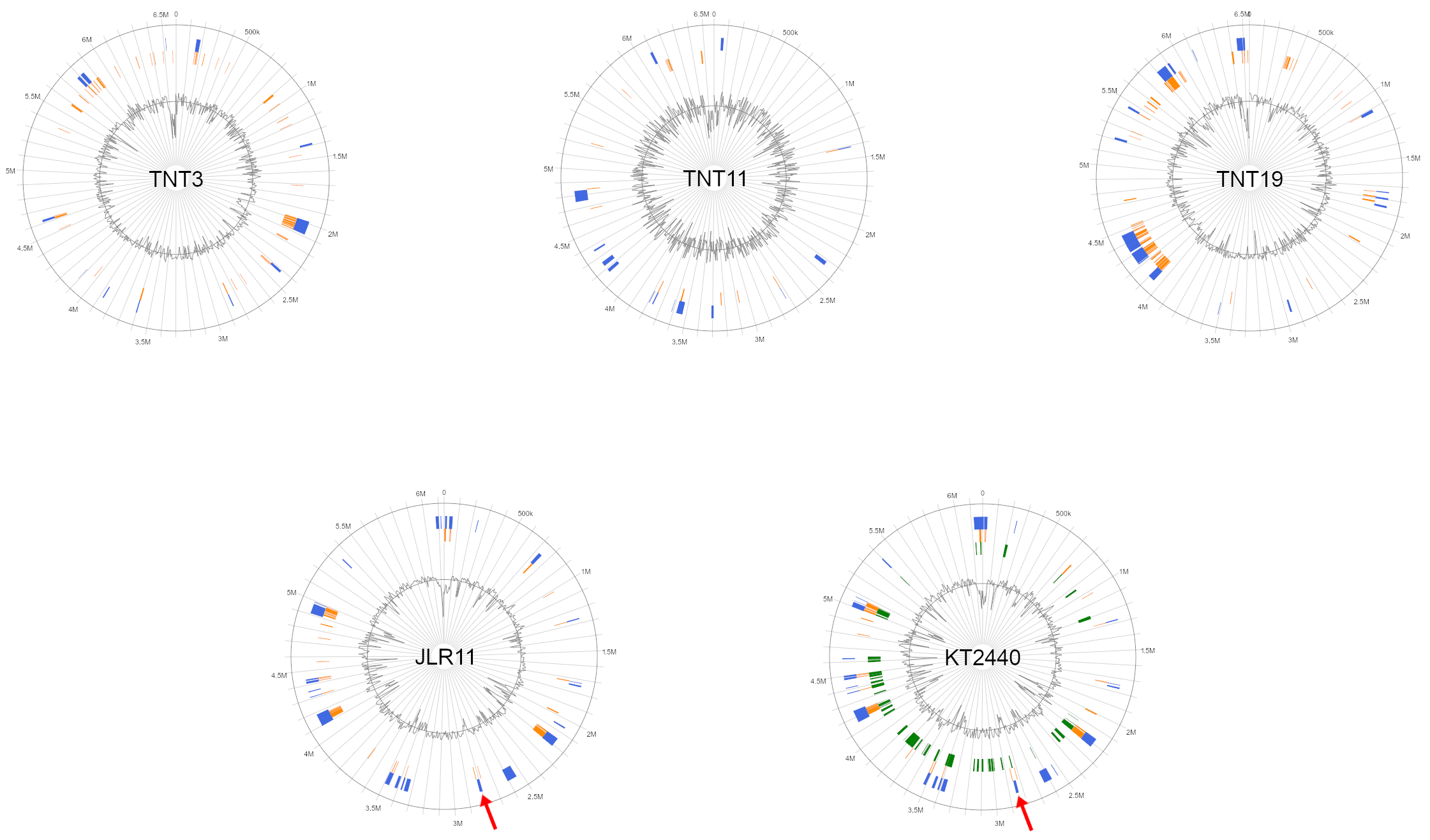

Supplement: Supplementary file 1 [file genes-13-01354-s001.zip › supplementary_figures/fig_S9.png]
